# The effects of age and dietary restriction on the tissue-specific metabolome of *Drosophila*

**DOI:** 10.1111/acel.12358

**Published:** 2015-06-18

**Authors:** Matthew J Laye, ViLinh Tran, Dean P Jones, Pankaj Kapahi, Daniel E L Promislow

**Affiliations:** 1 Buck Institute for Research on AgingNovato, CA, USA; 2 Division of Pulmonary Allergy & Critical Care Medicine, Department of Medicine, Emory UniversityAtlanta, GA, USA; 3 Department of Medicine, Clinical Biomarkers Laboratory, Emory UniversityAtlanta, GA, USA; 4 Department of Pathology, University of WashingtonSeattle, WA, USA; 5 Department of Biology, University of WashingtonSeattle, WA, USA

**Keywords:** *Drosophila*, aging, metabolome, metabolomics, bioinformatics, dietary restriction

## Abstract

Dietary restriction (DR) is a robust intervention that extends lifespan and slows the onset of age-related diseases in diverse organisms. While significant progress has been made in attempts to uncover the genetic mechanisms of DR, there are few studies on the effects of DR on the metabolome. In recent years, metabolomic profiling has emerged as a powerful technology to understand the molecular causes and consequences of natural aging and disease-associated phenotypes. Here, we use high-resolution mass spectroscopy and novel computational approaches to examine changes in the metabolome from the head, thorax, abdomen, and whole body at multiple ages in *Drosophila* fed either a nutrient-rich *ad libitum* (AL) or nutrient-restricted (DR) diet. Multivariate analysis clearly separates the metabolome by diet in different tissues and different ages. DR significantly altered the metabolome and, in particular, slowed age-related changes in the metabolome. Interestingly, we observed interacting metabolites whose correlation coefficients, but not mean levels, differed significantly between AL and DR. The number and magnitude of positively correlated metabolites was greater under a DR diet. Furthermore, there was a decrease in positive metabolite correlations as flies aged on an AL diet. Conversely, DR enhanced these correlations with age. Metabolic set enrichment analysis identified several known (e.g., amino acid and NAD metabolism) and novel metabolic pathways that may affect how DR effects aging. Our results suggest that network structure of metabolites is altered upon DR and may play an important role in preventing the decline of homeostasis with age.

## Introduction

Aging is a complex biological process that results in a gradual decline in physiological function and increases the prevalence of chronic diseases (Young, 1997; Kennedy *et al*., 2014). The effects of aging on survival and pathology can be reversed through genetic, pharmacological, and environmental perturbations (Fontana *et al*., 2010; Kapahi *et al*., 2010). The most reliable of these is dietary restriction (DR), which can extend lifespan in yeast, nematodes, fruit flies, rodents, and potentially nonhuman primates (reviewed in Fontana *et al*., 2010).

Researchers have proposed several biological processes to explain how DR extends lifespan. For instance, DR enhances autophagy, mitochondrial biogenesis, lipid metabolism, proteostasis, and stem cell function, while reducing oxidative stress and inflammation (Lopez-Lluch *et al*., 2006; Aris *et al*., 2013) and reversing the age-related decline in stem cell function and increase in inflammation (Horrillo *et al*., 2011; Cerletti *et al*., 2012). Genetically, the TOR, Sir 2, and insulin signaling pathways have been implicated in mediating the effects of DR on lifespan (Kapahi *et al*., 2004; Mair & Dillin, 2008).

The use of high-throughput ‘-omic’ approaches in genomewide expression studies demonstrates the power of systems biology approaches to increase our understanding of global changes with aging and anti-aging manipulations (Pletcher *et al*., 2005). Here, we extend these approaches and analyses through the use of high-resolution metabolomic profiling in several tissue types at several ages in response to AL and DR diets in the fruit fly, *Drosophila melanogaster*.

The metabolome is particularly valuable in studies on mechanisms of DR. In particular, the metabolome integrates information from multiple levels of organization, including the genome, the transcriptome, the proteome, the environment, and their interactions (Chan *et al*., 2010; Jones *et al*., 2012). Metabolic profiles vary with age in worms (Fuchs *et al*., 2010), flies (Sarup *et al*., 2012; Hoffman *et al*., 2014), mice (Tomas-Loba *et al*., 2013), marmosets (Soltow *et al*., 2010), and humans (Menni *et al*., 2013). Furthermore, recent studies suggest that DR might reverse the effects of aging on the metabolome (Avanesov *et al*., 2014). In light of these studies, it is clear that metabolomic profiling offers tremendous potential to understand the causes and consequences of aging (Mishur & Rea, 2012).

In addition to identifying effects of DR on levels of individual metabolites, we can also infer underlying mechanisms by identifying the effect of DR on metabolomic network structure. Such ‘differential correlation network’ approaches are relatively common in transcriptomic studies (e.g., Tesson *et al*., 2010). Numerous studies have shown that the underlying structure of molecular networks can shift dramatically under changing conditions, in particular, in the presence of disease (Amar *et al*., 2013). In fact, close analysis of network structure not only has diagnostic potential, but might also point toward specific mechanisms underlying causes of aging. For example, Southworth *et al*. (2009) used this approach to identify NFκB as a critical transcription factor in the loss of network connectivity with age in mice. Changes in network connectivity can occur regardless of changes in mean levels and thus include metabolites that would otherwise be ignored (Amar *et al*., 2013).

We used our metabolomic data to ask whether DR reverses age-related changes in the metabolome, and whether DR alters correlations between metabolites. We find that (i) DR significantly alters the levels of various individual metabolites; (ii) DR reverses age-related changes in a large fraction of the metabolome; (iii) although the mean levels of many metabolites are unchanged by diet, diet dramatically affects their correlation with other metabolites; and (iv) DR significantly alters the correlation structure of the metabolome network and slows or reverses age-related changes in network structure. Our results show that high-resolution metabolomic and network analyses serve as novel, powerful models which provide insights into the mechanisms that underlie the protective effects of DR on healthspan and lifespan in diverse organisms.

## Results

### Changes in the metabolome upon DR

**Fig 1:**
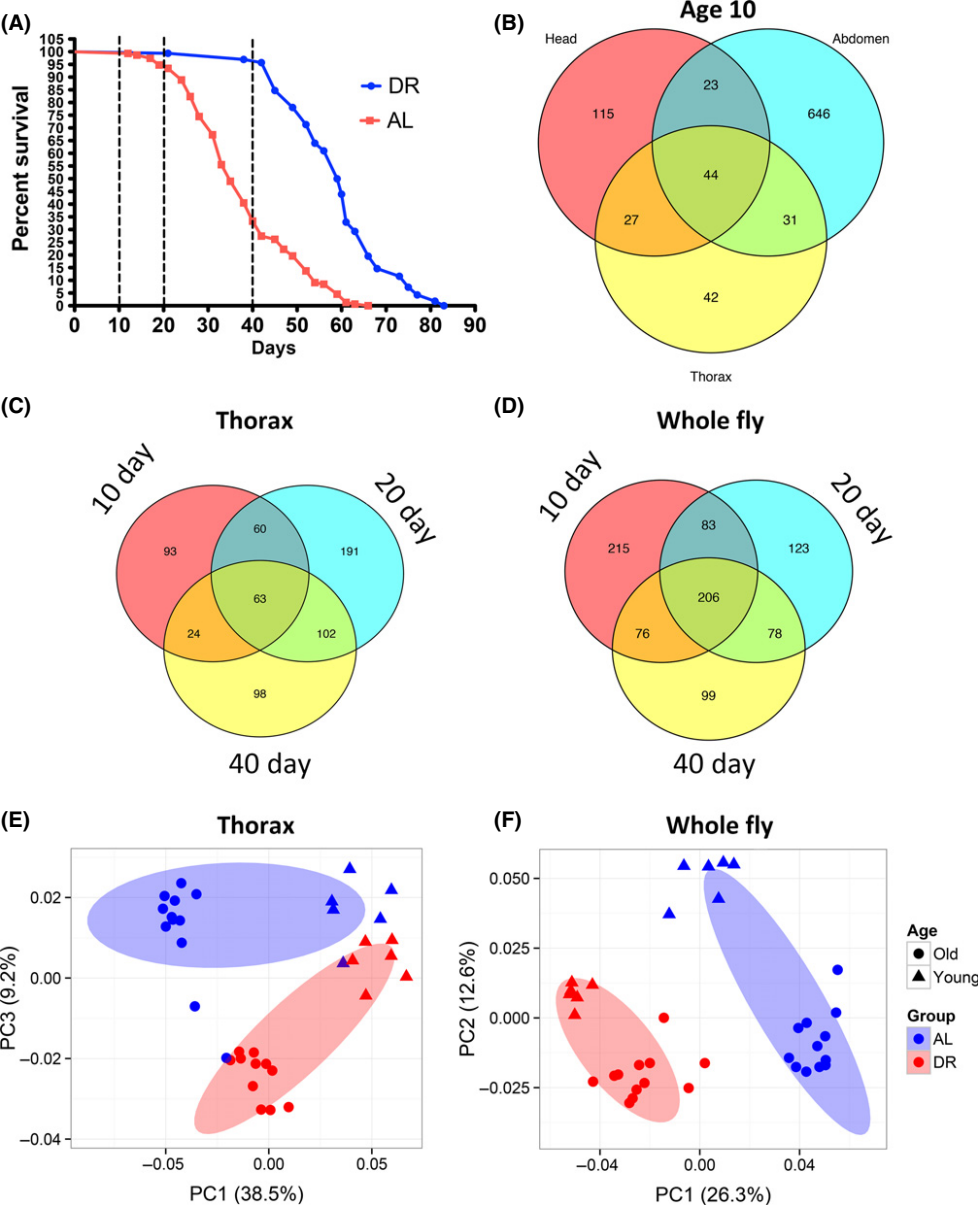
Multivariate and tissue-specific changes in the metabolome. (A) Lifespan curve of wild-type w^1118^ nonvirgin female flies fed a 0.5% yeast (DR) or 5% (AL) diet. Vertical dotted lines indicate ages at which samples were collected (biological replicates *n* = 6). Whole flies and thorax were collected at all three ages. Heads and abdomen were collected at 10 days of age only. (B–D) Venn diagram of numbers of metabolites altered by DR (simple linear model testing at *P* < 0.01, which is less than FDR = 0.1 in all cases) in head, abdomen, and thorax at 10 days of age (B) in the thorax (C) and whole fly (D) at 10, 20, and 40 days of age. (E–F) Principal component analysis of thorax (E) and whole fly (F) samples from young (10 days) and old (20 and 40 days combined) flies. Ellipses indicate 75% confidence interval.

As seen in many previous studies, we found that DR in *w*^*1118*^ female mated flies extended mean and median lifespan (Fig.1A). High-resolution nontargeted metabolomic analyses were carried out in 10-day-old *w*^*1118*^ flies in head, abdomen, and thorax separately. We found 928 metabolites that were significantly affected by DR at this age, of which 646 were unique to the abdomen, 115 unique to the head, and 42 unique to the thorax (Fig.1B).

Thorax and whole fly samples were collected at three ages, 10 days of age when neither AL nor DR fed flies have begun to die, 20 days of age when AL fed flies have just begun to die, and 40 days of age when DR fed flies start to die and AL fed flies are close to their median lifespan (Fig.1A). In thorax and whole fly samples, the number and overlap of metabolites that changed at the different ages is shown in a Venn diagram (Fig.1C,D). We found that between 10% (thorax) and 23% (whole fly) of all metabolites were affected by DR at one or more of the three sample ages (10, 20, and 40 days). Principal component analysis (PCA) clearly distinguished flies on different diets for both thorax (Fig.1E) and whole fly (Fig.1F) samples, in both young (10 days) and old (40 days) flies.

For each tissue- and age-specific list of metabolites affected by DR, we carried out metabolite set enrichment analysis (MSEA) using the *mummichog* program, which identifies and maps metabolites to pathways (Tables1 and 2) (Li *et al*., 2013). These samples included the four tissue types (head, abdomen, thorax, and whole body) from day 10 and two types (thorax and whole) from days 20 and 40 for a total of eight conditions. This comparison enabled us to identify metabolite pathways whose response to DR changed in a tissue- and/or age-specific manner.

**Table 1:** Metabolic sets decreased on DR

Metabolic Sets Decreased	Head	Abdomen	Thorax10	Thorax20	Thorax40	Whole10	Whole20	Whole40	Frequency
Amino acids	X	X	X	X	X	X	X	X	8
Beta-alanine betaine biosynthesis	X		X	X	X		X	X	6
S-adenosyl-L-methionine cycle	X			X	X		X	X	5
Choline biosynthesis III					X	X	X	X	4
Isoleucine biosynthesis from threonine		X		X		X	X		4
Methionine degradation I (to homocysteine)				X	X		X	X	4
Phenylethanol biosynthesis					X		X	X	3
Phosphatidylcholine biosynthesis					X		X	X	3
Serotonin and melatonin biosynthesis	X			X			X		3
Asparagine biosynthesis III	X					X			2
Citrulline-nitric oxide cycle				X	X				2
NAD Biosynthesis from 2-amino-3-carboxymuconate semialdehyde		X				X			2
Phenylalanine degradation I						X	X		2
Purine deoxyribonucleosides degradation							X	X	2
Tryptophan degradation to 2-amino-3-carboxymuconate semialdehyde		X		X					2
Valine degradation		X				X			2
2-methylbutyrate biosynthesis							X		1
4-hydroxyproline degradation I						X			1
5-aminoimidazole ribonucleotide biosynthesis I						X			1
Aspartate degradation II						X			1
Catecholamine biosynthesis							X		1
Dopamine degradation								X	1
Folate transformations					X				1
FormylTHF biosynthesis					X				1
Glutathione redox reactions I							X		1
Glutathione redox reactions II							X		1
Isoleucine degradation I								X	1
Proline degradation I						X			1
Spermidine biosynthesis								X	1
Spermine biosynthesis								X	1
TCA cycle variation III (eukaryotic)		X							1
Urate biosynthesis							X		1
Urate biosynthesis								X	1
Uridine-5′-phosphate biosynthesis		X							1
Uridine-5′-phosphate biosynthesis						X			1

**Table 2:** Metabolic sets increased on DR

Metabolic Pathways Increased	Head	Abdomen	Thorax10	Thorax20	Thorax40	Whole10	Whole20	Whole40	Frequency
Catecholamine biosynthesis				X		X	X	X	4
Lipoate biosynthesis and incorporation II		X				X	X	X	4
Degradation of purine ribonucleosides		X				X		X	3
Beta-alanine betaine biosynthesis							X	X	2
Dopamine degradation							X	X	2
FormylTHF biosynthesis I							X	X	2
Glycogen degradation I		X						X	2
Purine deoxyribonucleosides degradation						X		X	2
Tyrosine degradation I		X						X	2
4-hydroxyphenylpyruvate biosynthesis		X							1
Aerobic respiration – electron donors reaction list		X							1
Citrulline-nitric oxide cycle						X			1
NAD biosynthesis from 2-amino-3-carboxymuconate semialdehyde					X				1
Pentose phosphate pathway (nonoxidative branch)		X							1
Pentose phosphate pathway (oxidative branch)		X							1
Pentose phosphate pathway (partial)		X							1
Salvage pathways of adenine						X			1
Salvage pathways of guanine						X			1
Superpathway of gluconate degradation		X							1
Trehalose degradation II (trehalase)		X							1
Tryptophan degradation to 2-amino-3-carboxymuconate semialdehyde				X					1
UDP-galactose biosynthesis (salvage pathway from galactose using UDP-glucose)	X								1

The metabolite set for amino acids was most robustly altered, showing a decline in response to DR in every tissue and at every age. No other metabolic set was enriched in all eight conditions. However, in thorax and whole fly at 20 and 40 days of age, three metabolic sets, beta-alanine biosynthesis, S-adenosyl-L-methionine cycle, and methionine degradation I (to homocysteine) were downregulated upon DR. The abdomen had the greatest number of metabolites significantly affected by DR, and some metabolic sets were uniquely enriched in the abdomen. For example, TCA cycle variation and uridine-5′phosphate biosynthesis pathways were downregulated, while the pentose phosphate pathway, gluconate degradation, trehalose degradation, and UDP-galactose biosynthesis metabolic sets were increased uniquely in the abdomen (Tables1 and 2). Together, these data suggest that DR affects some metabolic pathways consistently across conditions, while other metabolic pathways are only altered in a single tissue or with age.

#### Validation of putative metabolites in an independent sample

To validate the changes in metabolites in our dataset, we conducted a metabolomic screen using the metabolomic services at Metabolon, which utilizes internal standards to validate metabolites. We then compared the Metabolon-generated list of validated metabolites with our putative metabolites (identified with the *mummichog* program) from 10-day-old flies fed either a DR or AL diet (Table S5). The diet effect on 29 metabolites that were matched by *m/z* with *mummichog* and independently by Metabolon was highly significantly correlated (*F*_1,27_ = 28.9, *P* = 1.1 × 10^−5^, Fig. S4). Among the metabolites that increased under DR in both experiments were carnitine, acetylcarnitine, and adenine, while those that decreased in both experiments included kynurenine, leucine, tryptophan, betaine, and pantothenate.

#### DR attenuates age-dependent changes in metabolites in old flies

**Fig 2:**
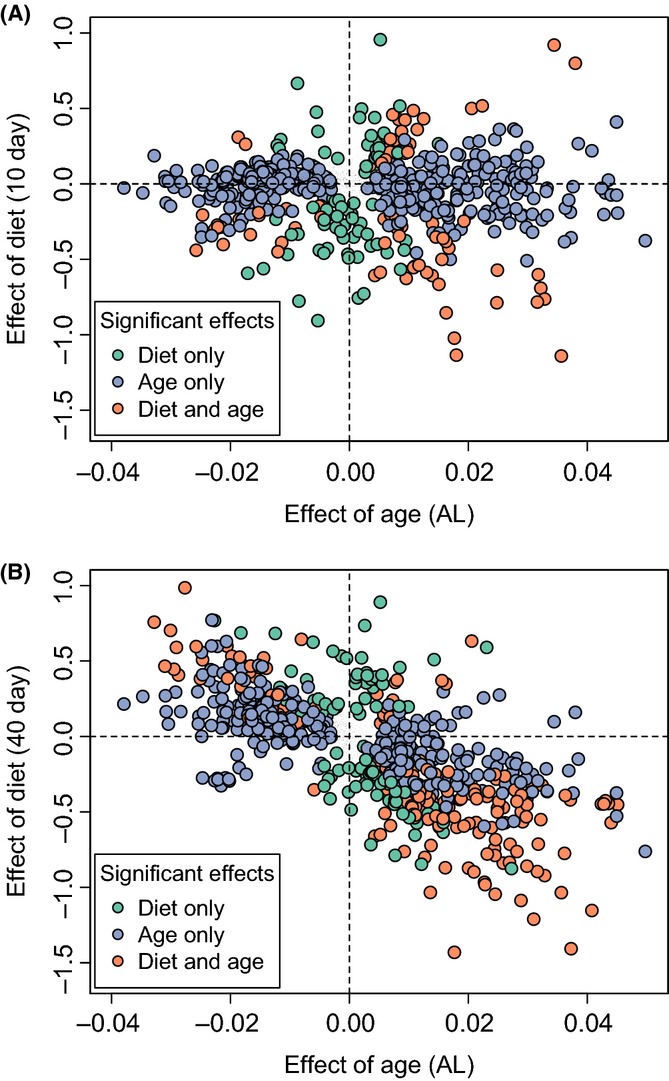
Effect of age and diet on the metabolome. Each point represents a metabolite. The axes show the β coefficient from the linear models of metabolite concentration vs. age (comparing 10 and 40 days under AL) on the *y*-axis and diet [comparing AL and DR at 10 days (A) or 40 days (B)] on the *x*-axis. Positive values represent metabolites that increase under DR conditions or with age. Metabolites shown are significantly (FDR = 0.025) altered by age (blue circles), diet (green circles), or both diet and age (orange circles) in the thorax. Metabolites in the top-left and bottom-right quadrants represent those for which DR reverses the effects of age.

We hypothesized that DR would reverse the effect of age on the metabolome as early as 10 days of age. In particular, we asked whether the effect of diet on each metabolite would reverse the effects of age on that metabolite. For example, if metabolite *i* increases with age, would DR *decrease* the level of that metabolite, effectively reversing the effect of age? This pattern is explored in Fig.2. Points representing metabolites for which diet reverses the effect of age would appear in the top-left and bottom-right quadrants. Those for which diet exacerbates the effects of age would appear in the top-right and bottom-left quadrants. At 20 and 40 days of age, we saw a dramatic DR-mediated reversal in the effects of age on metabolite abundance (Fig.2B, Fig. S2). However, at 10 days of age, we saw no relationship between diet-specific and age-specific changes in metabolite levels (Fig.2A).

#### Effect of DR on metabolite correlations

**Fig 3:**
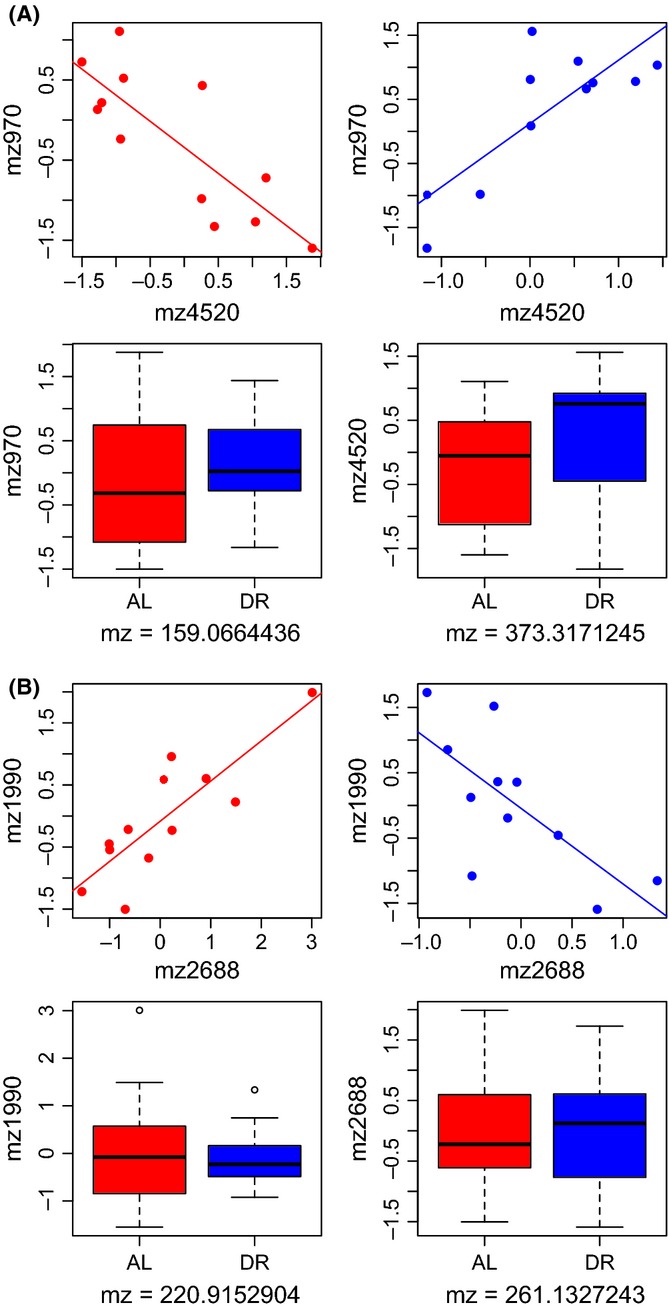
Pairs of thorax metabolites in (A) and (B) whose mean values are unaffected by diet (*P* > 0.05) but whose correlations are significantly different between DR and AL. The upper panels show the relationship between two metabolites under AL (red) and DR (blue) conditions. The correlations are significantly different in both cases (A: ANCOVA *F*_1,19_ = 34.7, *P* = 1.13 × 10^−5^; B: ANCOVA *F*_1,19_ = 24.8, *P* = 8.32 × 10^−5^). The lower panels show the effect of diet on the absolute levels of each metabolite (none are statistically significant).

Based on previous work in mice (Southworth *et al*., 2009) that identified changes in network connectivity with age, we hypothesized that for some metabolites, diet might not change the mean concentration across samples, but instead might influence their correlation with other metabolites. To this end, we sought pairs of metabolites, neither of which change mean values in response to changes in diet, but whose correlation coefficients with other each other did change significantly between AL vs. DR diets. We saw that differential correlation was relatively common and occurred in many ways. For example, some pairs of metabolites were positively correlated in DR and negatively correlated in AL, while other pairs showed the opposite trend. Representative pairs and examples of the different correlation patterns are in Fig.3 and Fig. S3.

#### DR changes network correlation connectivity

**Fig 4:**
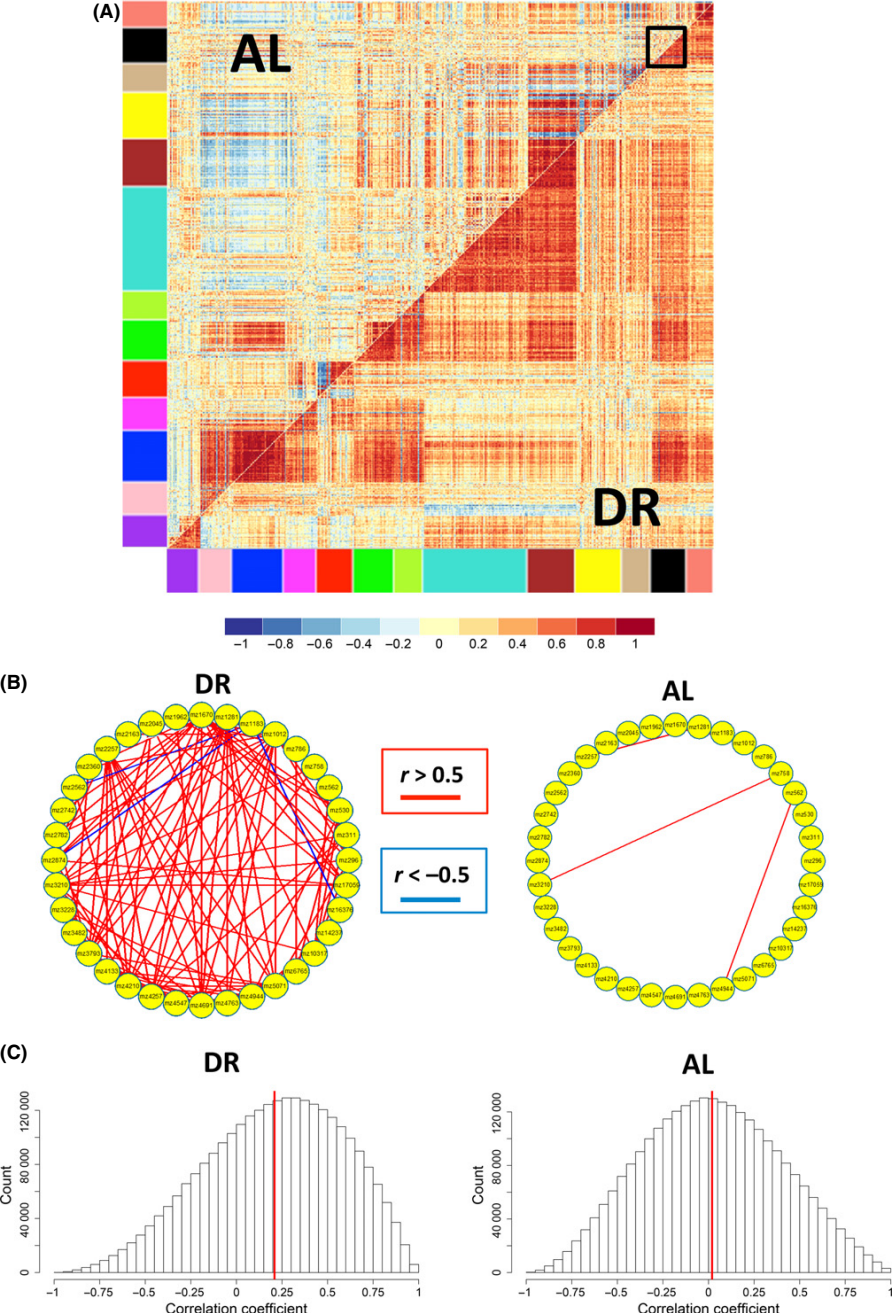
Differential coexpression analysis of all metabolites (ages 20 and 40, and thorax and whole fly combined, correcting for batch effects using combat). (A) Correlation matrix of metabolites significantly correlated (*n* = 550) in DR or AL, but not both diets. Modules of metabolites are indicated by the colored bars. The black box highlights the module of metabolites shown in detail in (B). (B) Detailed examination of the correlation network of metabolites in the black module from (A) in DR (left panel) and AL (right panel). Individual metabolites are represented by yellow circles, with positive correlations (*r* > 0.5) shown in red and negative correlations (*r* < −0.5) shown in blue. (C) Distribution of correlations between metabolites in DR (left panel) and AL (right panel) for thorax. The solid red line indicates the mean of all correlations. Across all metabolites in (A), correlations are significantly higher for DR than AL (*P* < 1e-16).

Motivated by the finding that diet affected the correlation pattern of pairs of metabolites, we then looked at metabolome-wide effects of diet on network structure, using differential coexpression ‘DiffCoEx’ analysis. We found striking differences in the correlation network of the metabolome between AL and DR conditions (Fig.4). Under DR conditions, correlations between metabolites trended toward more positive correlations compared with correlations under AL conditions (Fig.4C). Using DiffCoEx, we identify modules consisting of multiple metabolites that shared common, diet-specific, differential correlation patterns. We then asked whether each module was enriched for metabolites in specific metabolic pathways. Among the enrichment patterns that we identified (Tables S3 and S4), we found modules enriched for NAD biosynthesis, salvage pathway for guanine, and degradation of purine ribonucleotides (turquoise); arginine degradation and isoleucine biosynthesis (brown); and mannose degradation, glycogen degradation, and UDP-galactose biosynthesis (magenta). (We note that color assignment to specific modules is arbitrary for each analysis).

**Fig 5:**
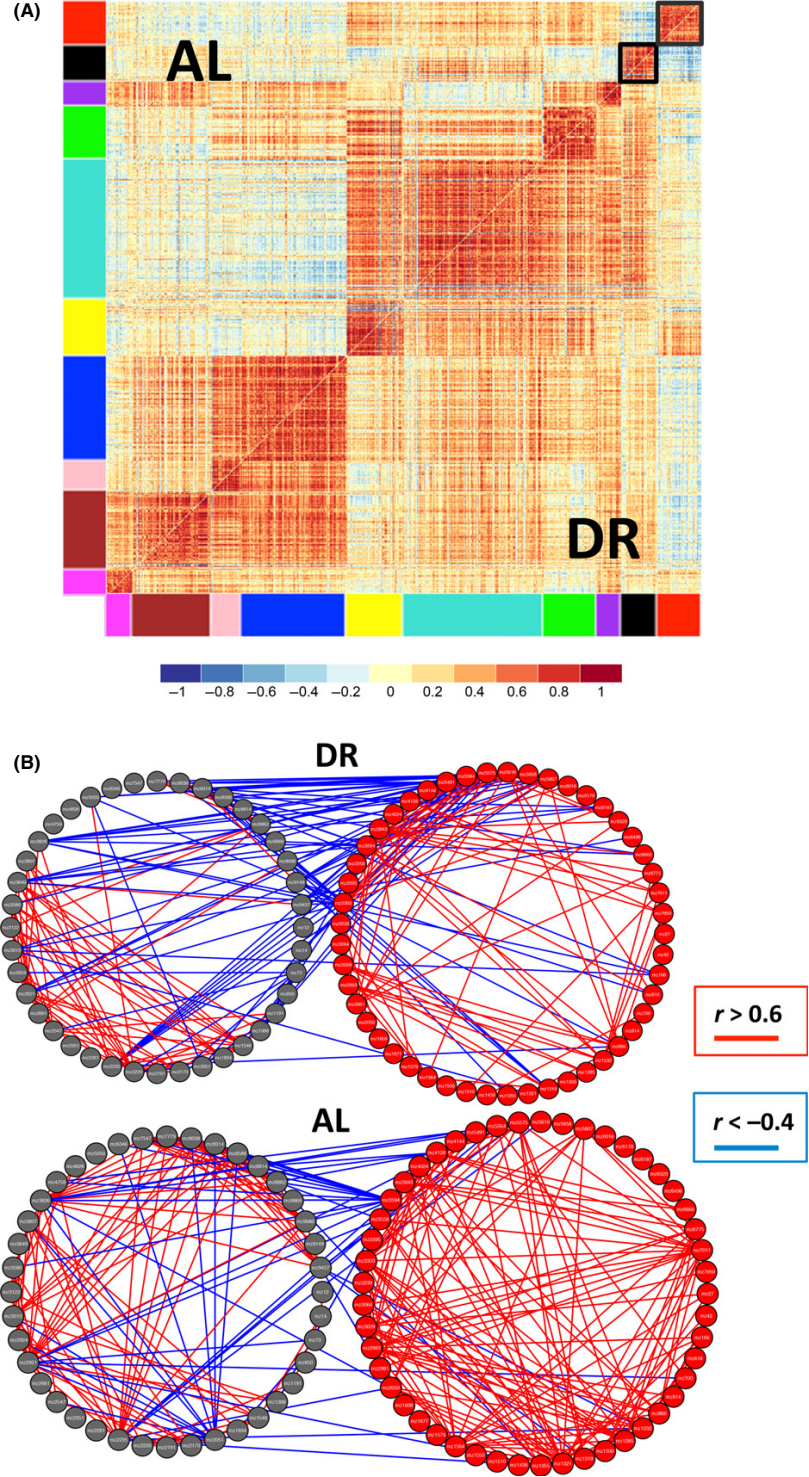
Similarity coexpression analysis of all metabolites (ages 20 and 40, and thorax and whole fly combined, corrected for batch effects using combat). (A) Correlation matrix of metabolites which show similarity correlation between AL and DR (*n* = 659). Modules of metabolites are indicated by the colored bars along bottom and left-hand side of matrix, with a scale indicating the strength of correlation between metabolites. The black boxes outlined in (A) are modules which are detailed in (B). (B) The correlation network of metabolites is examined in detail with the two modules represented by black circles and red circles from the correlation matrix modules in (A). DR (top) and AL (bottom). Individual metabolites are represented by black and red circles, respectively, with lines colored as in Fig.4B.

To complement our DiffCoEx analysis, we also identified groups of metabolites whose correlation network structure showed no change in comparison between both diet conditions, using an approach we call ‘SimCoEx’ (see Experimental procedures ). We identified some metabolic pathways that were enriched in the SimCoEx modules but not in the DiffCoEx modules, including glutamate metabolism (blue) and isoleucine degradation (red). To display the network connections in more detail, we present select DiffCoEx and SimCoEx modules, including the DiffCoEx black module (Fig.4B) and the SimCoEx black and red modules (Fig.5B). These metabolic pathways may provide insights into the key metabolites or metabolic reactions that cause aging in a similar manner that differential coexpression analysis in transcriptomic data has found transcription factors important in aging (Southworth *et al*., 2009).

#### Age changes network connectivity

**Fig 6:**
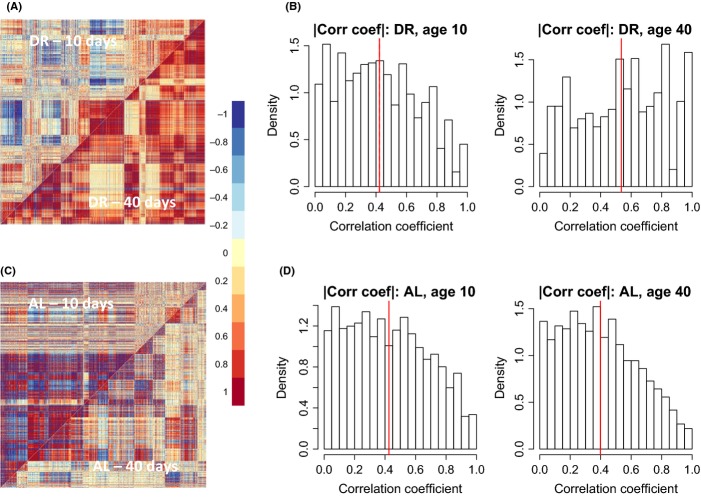
Differential coexpression analysis of thorax metabolites from AE column (A,C). Differential correlation matrix shows metabolites correlated between ages in flies fed a DR (A, *N* = 872) or AL (C, *N* = 845) diet. (B,D) The distribution of strength of correlations between metabolites at ages 10 (left) or 40 (right) under DR (B) and AL (D). The solid red line denotes the mean of all correlations. Note that DR attenuates the age-related decline in correlations among metabolites.

As we observed that DR reverses the effects of age on metabolite abundance, we hypothesized that changes in network structure with age are different in DR and AL fed flies. Accordingly, we used DiffCoEx to identify modules of differentially correlated metabolites at 10 days of age vs. 40 days of age for both DR and AL fed flies. The DiffCoEx figures (Fig.6A, C) suggest that under DR, correlation coefficient strength increases with age, while under AL conditions, the strength of correlations decreases with age. These diet-specific differences in age-related changes are reflected in histograms of the correlation coefficients (Fig.6B, D). Thus, it appears that DR attenuates the age-related decline in the metabolome-wide correlations among metabolites.

#### Similar results were obtained with two different columns

We analyzed metabolite profiles for samples run on two kinds of column—an anionic exchange (AE) column and a nonpolar (C18) column. The patterns we found did not differ between columns. For simplicity, the AE column results were presented. Analyses of metabolite profiles from the C18 column are presented in the supplemental figures (Fig. S1–S3) and Tables (S1–S4).

## Discussion

Over the past 70 years, researchers have put substantial effort into the study of mechanisms by which DR extends lifespan (Mair & Dillin, 2008; Niccoli & Partridge, 2012). Despite this work, we know little about the functional mechanisms that determine how and why DR extends healthspan and lifespan. This gap in our knowledge motivated us to focus on the effects of DR on the metabolome, which includes the building blocks of so many functional pathways in an organism. Previous studies have found that DR alters not just physiological traits, such as starvation resistance (Katewa *et al*., 2012), mitochondrial activity (Zid *et al*., 2009; Katewa *et al*., 2012), and spontaneous activity (Parashar & Rogina, 2009), but also the transcriptome (Pletcher *et al*., 2002; Whitaker *et al*., 2014). The work presented here brings needed attention to the metabolome.

In mice and other vertebrate species, studies hint at the possibility that DR might influence lifespan or healthspan in part through preventing age-related changes in the metabolome. For example, De Guzman *et al*. (2013) found 77 of 7000 serum features that differed between young and old AL mice, 15 of which were attenuated by DR. In dogs, Richards *et al*. (2013) found the plasma metabolome differ with age in both DR and AL fed animals, but it was not clear from their study whether DR attenuated the age-related change in the metabolome. Finally, a study in Rhesus macaques found that age and DR alter the plasma metabolome and may prevent age-related changes in the metabolome (see Table 3 in Rezzi *et al*., 2009). While these studies demonstrate the power of metabolomic assays in DR studies, they are limited by assaying relatively few metabolites, and in some cases, by a study design that includes young AL animals but not young DR animals.

A recent metabolomic study by Avanesov *et al*. (2014) overcomes some of these shortcomings with the use of two different diets and high-resolution metabolomic profiles for numerous ages. Their results suggest that a diet that extends longevity shifts both the transcriptome and the metabolome toward a younger state, similar to our findings at 20 and 40 days of age. Other similarities include separation by age using principle component analysis and identification of similar but not identical enriched metabolite sets. There are some important differences between this work and our own. Our work used thorax and whole flies for all time points and included heads and abdomen at 10 days of age to look at tissue-specific effects of DR. In contrast, Avanesov *et al*. (2014) only used whole flies. Other methodological differences exist, including different mass spectrometry protocols (different columns for separation) and different fly strains (*w*^*1118*^ vs. *Canton S*). The main analytical difference is our inclusion of network analysis to determine the age and diet effect on correlations between networks; however, we also used different programs (*mummichog* vs. MetaboAnalyst) to identify metabolites and map them to specific metabolic pathways that may be enriched. Thus, direct comparisons between the two studies should be interpreted carefully.

Our metabolite set enrichment analysis with *mummichog* suggests numerous interesting avenues for future research. Not surprisingly, given that the DR approach used here consisted of protein restriction, we saw a strong and consistent decrease in amino acids in all tissues of DR relative to AL flies. Interestingly, in both thorax and whole fly at 20 and 40 days, we saw downregulation of the S-adenosyl-L-methionine cycle pathway. The S-adenosyl-L-methionine pathway provides the cell with methyl groups for methylation of nucleotides and proteins, a pathway implicated in age-related diseases (Park *et al*., 2012). The universal methyl donor, S-adenosyl-L-methionine synthetase, has been tied to lifespan in worms (Hansen *et al*., 2005) and flies (Lin *et al*., 2005). A second potential role for the S-adenosyl-L-methionine pathway to improve health is through the interaction of methionine and AMPK (Cabreiro *et al*., 2013). AMPK activation increases lifespan in worms, flies, and rodents and is the target of the antidiabetic drug metformin. In worms at least, metformin requires the metabolite methionine to have its anti-aging effects (Cabreiro *et al*., 2013), but whether the same interaction is present in other animal models is unknown. Furthermore, other intermediates within the methionine metabolism pathway, such as homocysteine, are implicated in aging or age-related diseases. For instance, homocysteine accelerates senescence of primary endothelial cells with increasing passage number (Zhang *et al*., 2015) and high levels of homocysteine are associated with cognitive decline and Alzheimer’s disease (reviewed Kronenberg *et al*., 2009). One possible reason for this downregulation will be reduced amino acids in the diet and thus a reduced need for amino acid degradation pathways.

Our tissue-specific analysis suggests that DR may regulate different metabolic processes in different tissues. For example, only in the abdomen did we see evidence of a DR-mediated increase in the pentose phosphate pathway and aerobic transport pathways. One important role of the pentose phosphate pathway is to generate NADPH for reductive biosynthesis of metabolites such as fatty acids. The *Drosophila* abdomen is both a site for *de novo* lipogenesis (Palm *et al*., 2012) and lipid export as the abdomen contains lipid-carrying carnitine moieties (Chintapalli *et al*., 2013). We have shown that the turnover of lipid in muscle is critical for beneficial effects of DR on lifespan and metabolism (Katewa *et al*., 2012). While it was beyond the scope of the current study to look at the effects of DR on all tissues, Chintapalli *et al*. (2013) performed metabolomics and lipidomics on ten different fly tissues under a single diet and show each tissue has a specific metabolite profile. A more complete analysis of tissue-specific responses to DR and age should greatly increase our understanding of the underlying mechanisms by which DR alters the metabolome and ultimately increases healthspan and lifespan.

While additional tissue-specific data will prove insightful, so will new analytical and computational approaches to existing data. For example, rather than focus just on changes in mean values, recent systems biology studies highlight the value of looking at correlations between variables. This has most commonly taken the form of transcriptome data, associating changes in network structure with a variety of diseases (Amar *et al*., 2013). Perhaps most relevant to the work we have presented here, Southworth *et al*. (2009) found that correlation coefficients among transcripts measured in 13 different mouse tissues declined with age.

While rare in metabolomic studies, those that have used this approach do provide novel information into biochemical and metabolic regulation. For example, Muller-Linow *et al*. (2007) found that the structure of the metabolite correlation network in *Arabidopsis thaliana* varied with time of day and that networks that were further apart in time were further apart in structure. Until now, differential metabolome network analyses have been limited to studies in plants (e.g., DiLeo *et al*., 2011; Fukushima *et al*., 2011). To our knowledge, ours is the first study to use differential coexpression analysis to look at diet- and age-related changes in the metabolome captured with high-resolution metabolomics.

Differential coexpression analysis identified numerous modules of metabolites whose correlation patterns changed in tandem between AL and DR conditions. These observations, combined with the well-known result that DR reduces age-related mortality rate and slows age-related declines in function, led us to ask how diet influenced age-related changes in network correlation. As anticipated, we found a loss of network correlation under *ad lib* conditions (consistent with Southworth *et al*., 2009), but if anything, DR flies showed greater network correlation at older ages (Fig.6). In both DR and AL conditions, the changes in network connectivity occurred in a modular, nonlinear fashion, suggesting a biologically coordinated mechanism. However, unlike Southworth *et al*.’s (2009) result, it is unlikely that transcription factors can account for changes in correlations between metabolites. We hypothesize that these changes reflect alterations in flux through specific metabolic pathways, but the underlying details are likely quite complex. We would also note that our network is based on relatively few samples. Increased sample size in future studies will be critical to develop a more robust portrait of the effects of diet and age on network structure.

As Muller-Linow *et al*. (2007) point out, one can often observe pairs of metabolites that are correlated across samples, but which are apparently unrelated in a map of known metabolic reactions. Future studies with targeted metabolomic approaches should allow us to measure the degree of overlap (or lack thereof) for metabolites that show diet- and age-specific changes in correlation structure. Testing hypotheses related to these differential correlations is not straightforward. If we observe levels in a single metabolite that are associated with a trait of interest, we can try to increase or decrease levels of that metabolite through genetic (Eisenberg *et al*., 2014) or pharmacological (Wang *et al*., 2014) means. More challenging still, it remains to be seen whether we can alter age-related changes in metabolite correlations by feeding two or more metabolites to flies simultaneously, or altering expression patterns of multiple enzymes associated with metabolites whose correlations change with age.

We would stress here that we do not yet know the significance of these correlations, but they may be a reflection of organismal homeostasis. Using the *mummichug* program (Li *et al*., 2013), we were able to identify enriched metabolic pathways in groups of metabolites not only whose mean values changed with diet, but also whose correlation network changed with diet (Fig.3). For example, within turquoise module in Fig.3, we identified enrichment for many metabolic pathways. Some of these pathways are important for amino acid metabolism including glutamate biosynthesis, glutamine degradation, arginine degradation, proline biosynthesis, and serine biosynthesis, while other pathways are important for energy metabolism, including oxidative ethanol degradation, pentose phosphate pathways (oxidative branch), and glycolysis. Changes in correlation patterns provide additional insights into the metabolome compared to changes in just the level of metabolites. For instance, L-carnitine biosynthesis is only enriched in DiffCoEx analysis, and carnitines have been shown to be altered with aging (Costell *et al*., 1989; Hoffman *et al*., 2014) and dietary restriction in multiple species, including humans (Redman *et al*., 2011).

The biological significance of changes in individual correlation coefficients and in the larger network structure is not entirely known yet. We speculate that a more highly correlated metabolome could reflect improved homeostasis and more tightly regulated metabolism. For example, in response to cold stress, *Drosophila* selected for cold adaptability have a more robust metabolic network than flies selected for cold susceptibility (Williams *et al*., 2014). Similarly, DR fed flies are more stress resistant. Many metabolic pathways occur in unique, but interdependent subcellular compartments. A higher degree of correlation might reflect a metabolome that is more stoichiometrically balanced, leading to higher efficiency of metabolic reactions. Thus, higher metabolic network connectivity may indicate an improved ability to respond to various environmental stressors.

As with any metabolomic analysis, there are some limitations to our study. First, there is not yet a curated fly metabolome. Of the several thousand metabolites in our dataset, only 14–21% were assigned putative chemical definitions in *mummichog*. Metabolic set enrichment analysis is limited by the metabolic coverage used to create the pathways, and likely represents only a fraction of the metabolic pathways altered by DR. Furthermore, food- or bacterial-derived metabolites may act as confounding factors (Corby-Harris *et al*., 2007). The oldest flies sampled were 40 days old, and thus, we might have missed important metabolite interactions that occur at older ages. We flash-froze our samples to best reflect the *in vivo* metabolome. This approach means that individual ‘tissue-specific’ samples actually combine many different specialized organs and tissues. Lastly, in this study, we used a widely available laboratory strain, *w*^1118^, but we know from recent studies that there are dramatic differences in metabolomic profiles among genotypes (Hoffman *et al*., 2014), and different genotypes are also likely to respond to DR differently (Forster *et al*., 2003).

Our work expands upon the observation that DR reverses transcriptomic changes with age to reversal of metabolomic changes with age. We also demonstrate the utility of using differential coexpression analysis in metabolomic studies and speculate that a common stress-resistant highly connected metabolome may exist in flies. Future work using DR in combination with both metabolomic and transcriptomic approaches will further tighten the molecular mechanisms that link metabolites with organismal phenotype.

## Experimental procedures

### Animals and rearing protocols

*w*^1118^ flies were maintained on standard stock food in a fly room kept at 25 °C and 60% humidity. All studies used female mated flies as they show the biggest difference in lifespan in response to changes in yeast in the diet. Flies were treated identically for lifespan and metabolomic analysis. Twenty-five adult nonvirgin female flies, 0–3 days old, were transferred to vials containing either 5% (w/v) yeast extract (herein referred to AL food) or 0.5% (w/v) yeast extract (herein referred to as DR food) as previously described (Katewa *et al*., 2012). Flies were transferred to fresh food three times per a week at which time dead flies were counted and removed. Median lifespans for DR (*n* = 164) and AL (*n* = 153) were 59.5 and 35 days, respectively.

Twenty-four hours prior to sample collection, flies were transferred to fresh food. Samples were collected at 10, 25, and 40 days following placement on different diets. These days were selected to represent a young, middle-aged, and median lifespan in AL fed flies (Fig.1A). To collect the different tissues, flies were snap-frozen in liquid nitrogen and dissected on dry ice at the same time of day, 1500 h. For all three ages, each sample (*n* = 6) contained 15 thoraces, 15 abdomens, or three whole flies, while 10-day samples also had heads collected (*n* = 6, 40–50 heads per sample) totaling ∼3 mg of tissue. Within each treatment group, the six biological replicates consisted of populations of flies reared and raised in distinct bottles and vials.

### Metabolomic assays

Sample preparation and analysis were carried out as described previously (Hoffman *et al*., 2014). For each biological replicate, acetonitrile extracts of the tissue were analyzed in a dual column [C18 and anion exchange (AE)] chromatography–mass spectrometry (LC-MS) platform. Samples were fractionated after electrospray ionization and subsequently detected using a Fourier transform mass spectrometer. Non-annotated mass/charge (m/z) features, column retention time, and ion intensity were collected as data. Fly standards were derived from a pooled reference sample stored as a large number of aliquots at −80 °C and run alongside experimental samples to allow comparison of analytic behavior over long periods of time. Data from the pooled reference samples were used for quality control (i.e., to ensure relative consistency among identical samples within days) and for quality assurance (i.e., to ensure consistent results between days), but were not used in downstream statistical analysis.

### Statistical analysis

All analyses described below were carried out using the open source software package R (Team 2013).

### Quality control

Metabolomic data consisted of separate files with features and counts from the AE and C18 columns. The C18 column consisted of 34 771 features distinguishable by mass/charge ratio (m/z) and column retention time, from a minimum m/z = 85.0279778 to a maximum of m/z = 1976.50254. The AE column included 17 617 features, from m/z = 86.0605321 to m/z = 1738.34568. Including all tissue types, diets, and biological and technical replicates, feature counts were available for 321 (C18) and 324 (AE) samples. Feature counts were log-transformed to approximate Gaussian distribution, and outlier samples were detected using the flashClust function in R, with a threshold cutoff *Z*_*k*_ = −3. One low yeast concentration thorax sample at age 40 was removed from the AE column data.

We then removed any metabolomic features with a signal-to-noise ratio (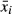/σmz_(*i*).tech_) < 20, where 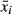 is mean of the *i*^th^ analyte and σ_mz.tech_ is the average standard deviation of each technical replicate for the *i*^th^ analyte. After taking the mean values of the technical replicates for all remaining features, we then created separate files by tissue type and, within each file, eliminated any features with more than 10% missing samples, imputing the remaining missing values using the *emArray* method in the LSimpute package (Bo *et al*., 2004). Finally, unless otherwise noted, all features were normalized to have a mean of 0, but were not rescaled to have unit variance.

#### Multivariate analysis

For each tissue type, we used principal component analysis, as implemented in R using the ord function, to determine whether there was a metabolome-wide signature of diet in the fly.

#### Univariate analysis

Given the large number of metabolites tested, it is necessary to correct for potentially large table-wise error, with a high risk of false positives. In all univariate analysis, we adjusted *P*-values using the false discovery rate correction with FDR = 0.05 unless otherwise noted. For each tissue type, we used a simple linear model to test for significant effects of diet on metabolic feature concentration.

For whole body- and thorax-specific data, samples were obtained at three different ages. For these data, we wanted to know the relationship between the effect of diet vs. the effect of age on feature concentration. Effect size was defined as the slope of the lines from the model

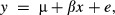
1

where *y* is the concentration of the metabolic feature, μ is the mean, *x* is age or diet, and β is the slope of the relationship between *x* and *y*. Due to limited sample size, we pooled thorax and whole body data, removing any batch effects due to differences between thorax and whole body using the ComBat function in R’s sva package (Leek *et al*., 2012). In comparing the relative impact of diet and age simultaneously, to avoid confounding effects, we tested for the effects of diet on just one age at a time and tested for the effects of age on just the high yeast concentration diet.

To determine the joint effect of diet and age on individual features, we plotted the β values (Eqn 1) for diet against the β values for age. Features for which diet reversed the effects of age would be expected to have opposite sign values for β.

#### Network structure

In addition to looking at the main effects of diet on the concentration of individual metabolites, here we also want to identify groups of metabolites whose overall correlation network is influenced by diet. To do so, we apply the differential coexpression package, DiffCoEx (Tesson *et al*., 2010), which takes advantage of the subroutines that make up part of the Weighted Gene Correlation Network Analysis package (WGCNA, Langfelder & Horvath, 2008) in R. WGCNA takes the correlation matrix (aka adjacency matrix, **A**) for a set of elements and then applies a clustering algorithm to identify clusters of similarly correlated elements. The package identifies modules of strongly correlated elements within a broader network. DiffCoEx takes two such modules, **A** and **A**′, made up of the same set of features measured in multiple samples, but across two different environments E and E′. We can construct a new ‘difference matrix’ 

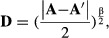
2which is a function of the two original matrices, where β is a scaling coefficient (Langfelder & Horvath, 2008). We can then carry out clustering analysis on **D** to identify groups of metabolites with common environment-specific changes in correlation network. We identified groups of metabolites that are significantly *preserved* across environments. To do this, we defined a ‘constancy matrix’,

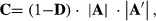
3on which we could carry out clustering analysis to reveal groups of metabolites that show relative constancy in the correlation network. Metabolites correlated between ages were normalized using the ‘normalize.quantiles’ function in bioconductor.

#### Metabolite set enrichment analysis

For both main effect and network analysis, to determine whether groups of metabolites that shared common responses were enriched for particular metabolic pathways, we used the program *Mummichog* (Li *et al*., 2013). Written in Python, *Mummichog* is a freely available program that provides putative matches to metabolites based on mass/charge ratios, and carries out the metabolomic equivalent of gene set enrichment analysis.

See the Supplemental Material for Experimental Details on the Metabolon Data Analysis.

## Supporting Information

**Fig. S1** Data from C18 column.

**Fig. S2** Effect of age and diet on the metabolome in C18 column separated metabolites.

**Fig. S3** Pairs of thorax metabolites whose mean values are unaffected by diet (*P* > 0.05) but whose correlations are significantly different between DR and AL.

**Fig. S4** (A) Correlation between overlapping metabolites from the targeted Metbolon approach and putatively matched metabolites by *mummichog* in the non-targeted high resolution approach (referred to as ‘Jones’) for the diet effect in 29 metabolites.

**Data S1** Metabolon Data Analysis. **Table S1** Metabolic Sets Decreased on DR from Metabolites Run on the C18 column. **Table S2** Metabolic Sets Enriched on DR from C18 Column. **Table S3** Metabolic Path Enrichment for Modules in DiffCoEx. **Table S4** Metabolic Sets Enriched from SimCoEx Analysis. **Table S5** Normalized Metabolite Levels from Metabolon Data Set.

## References

[b1] Amar D, Safer H, Shamir R (2013). Dissection of regulatory networks that are altered in disease via differential co-expression. PLoS Comput. Biol.

[b2] Aris JP, Alvers AL, Ferraiuolo RA, Fishwick LK, Hanvivatpong A, Hu D, Kirlew C, Leonard MT, Losin KJ, Marraffini M, Seo AY, Swanberg V, Westcott JL, Wood MS, Leeuwenburgh C, Dunn WA (2013). Autophagy and leucine promote chronological longevity and respiration proficiency during calorie restriction in yeast. Exp. Gerontol.

[b3] Avanesov AS, Ma S, Pierce KA, Yim SH, Lee BC, Clish CB, Gladyshev VN (2014). Age- and diet-associated metabolome remodeling characterizes the aging process driven by damage accumulation. eLife.

[b4] Berrougui H, Khalil A (2009). Age-associated decrease of high-density lipoprotein-mediated reverse cholesterol transport activity. Rejuvenation Res.

[b5] Bo TH, Dysvik B, Jonassen I (2004). LSimpute: accurate estimation of missing values in microarray data with least squares methods. Nucleic Acids Res.

[b6] Cabreiro F, Au C, Leung KY, Vergara-Irigaray N, Cocheme HM, Noori T, Weinkove D, Schuster E, Greene ND, Gems D (2013). Metformin retards aging in *C. elegans* by altering microbial folate and methionine metabolism. Cell.

[b7] Cerletti M, Jang YC, Finley LW, Haigis MC, Wagers AJ (2012). Short-term calorie restriction enhances skeletal muscle stem cell function. Cell Stem Cell.

[b8] Chan EK, Rowe HC, Hansen BG, Kliebenstein DJ (2010). The complex genetic architecture of the metabolome. PLoS Genet.

[b9] Chintapalli VR, Al Bratty M, Korzekwa D, Watson DG, Dow JA (2013). Mapping an atlas of tissue-specific *Drosophila melanogaster* metabolomes by high resolution mass spectrometry. PLoS ONE.

[b10] Corby-Harris V, Pontaroli AC, Shimkets LJ, Bennetzen JL, Habel KE, Promislow DEL (2007). Geographical distribution and diversity of bacteria associated with natural populations of *Drosophila melanogaster*. Appl. Environ. Microbiol.

[b100] Costell M, O’Connor JE, Grisolia S (1989). Age-Dependent Decrease of Carnitine Content in Muscle of Mice and Humans. Biochemical and biophysical research communications.

[b11] De Guzman JM, Ku G, Fahey R, Youm YH, Kass I, Ingram DK, Dixit VD, Kheterpal I (2013). Chronic caloric restriction partially protects against age-related alteration in serum metabolome. Age (Dordr).

[b12] DiLeo MV, Strahan GD, den Bakker M, Hoekenga OA (2011). Weighted Correlation Network Analysis (WGCNA) applied to the tomato fruit metabolome. PLoS ONE.

[b13] Eisenberg T, Schroeder S, Andryushkova A, Pendl T, Kuttner V, Bhukel A, Marino G, Pietrocola F, Harger A, Zimmermann A, Moustafa T, Sprenger A, Jany E, Buttner S, Carmona-Gutierrez D, Ruckenstuhl C, Ring J, Reichelt W, Schimmel K, Leeb T, Moser C, Schatz S, Kamolz LP, Magnes C, Sinner F, Sedej S, Frohlich KU, Juhasz G, Pieber TR, Dengjel J, Sigrist SJ, Kroemer G, Madeo F (2014). Nucleocytosolic depletion of the energy metabolite acetyl-coenzyme a stimulates autophagy and prolongs lifespan. Cell Metab.

[b14] Fontana L, Partridge L, Longo VD (2010). Extending healthy life span–from yeast to humans. Science.

[b15] Forster MJ, Morris P, Sohal RS (2003). Genotype and age influence the effect of caloric intake on mortality in mice. FASEB J.

[b16] Fuchs S, Bundy JG, Davies SK, Viney JM, Swire JS, Leroi AM (2010). A metabolic signature of long life in *Caenorhabditis elegans*. BMC Biol.

[b17] Fukushima A, Kusano M, Redestig H, Arita M, Saito K (2011). Metabolomic correlation-network modules in *Arabidopsis* based on a graph-clustering approach. BMC Syst. Biol.

[b18] Hansen M, Hsu AL, Dillin A, Kenyon C (2005). New genes tied to endocrine, metabolic, and dietary regulation of lifespan from a *Caenorhabditis elegans* genomic Rnai screen. PLoS Genet.

[b19] Hoffman JM, Soltow QA, Li S, Sidik A, Jones DP, Promislow DEL (2014). Effects of age, sex, and genotype on high-sensitivity metabolomic profiles in the fruit fly, *Drosophila melanogaster*. Aging Cell.

[b20] Horrillo D, Sierra J, Arribas C, Garcia-San Frutos M, Carrascosa JM, Lauzurica N, Fernandez-Agullo T, Ros M (2011). Age-associated development of inflammation in Wistar rats: effects of caloric restriction. Arch. Physiol. Biochem.

[b21] Jones DP, Park Y, Ziegler TR (2012). Nutritional metabolomics: progress in addressing complexity in diet and health. Annu. Rev. Nutr.

[b22] Kapahi P, Zid BM, Harper T, Koslover D, Sapin V, Benzer S (2004). Regulation of lifespan in *Drosophila* by modulation of genes in the TOR signaling pathway. Curr. Biol.

[b23] Kapahi P, Chen D, Rogers AN, Katewa SD, Li PW, Thomas EL, Kockel L (2010). With tor, less is more: a key role for the conserved nutrient-sensing TOR pathway in aging. Cell Metab.

[b24] Katewa SD, Demontis F, Kolipinski M, Hubbard A, Gill MS, Perrimon N, Melov S, Kapahi P (2012). Intramyocellular fatty-acid metabolism plays a critical role in mediating responses to dietary restriction in *Drosophila melanogaster*. Cell Metab.

[b25] Kennedy BK, Berger SL, Brunet A, Campisi J, Cuervo AM, Epel ES, Franceschi C, Lithgow GJ, Morimoto RI, Pessin JE, Rando TA, Richardson A, Schadt EE, Wyss-Coray T, Sierra F (2014). Geroscience: linking aging to chronic disease. Cell.

[b26] Kronenberg G, Colla M, Endres M (2009). Folic acid, neurodegenerative and neuropsychiatric disease. Curr. Mol. Med.

[b27] Langfelder P, Horvath S (2008). WGCNA: an R package for weighted correlation network analysis. BMC Bioinformatics.

[b28] Leek JT, Johnson WE, Parker HS, Jaffe AE, Storey JD (2012). The sva package for removing batch effects and other unwanted variation in high-throughput experiments. Bioinformatics.

[b29] Li S, Park Y, Duraisingham S, Strobel FH, Khan N, Soltow QA, Jones DP, Pulendran B (2013). Predicting network activity from high throughput metabolomics. PLoS Comput. Biol.

[b30] Lin MJ, Tang LY, Reddy MN, Shen CK (2005). DNA methyltransferase gene dDNMT2 and longevity of *Drosophila*. J. Biol. Chem.

[b31] Lopez-Lluch G, Hunt N, Jones B, Zhu M, Jamieson H, Hilmer S, Cascajo MV, Allard J, Ingram DK, Navas P, de Cabo R (2006). Calorie restriction induces mitochondrial biogenesis and bioenergetic efficiency. Proc. Natl Acad. Sci. USA.

[b32] Mahoney LB, Denny CA, Seyfried TN (2006). Caloric restriction in C57BL/6J mice mimics therapeutic fasting in humans. Lipids Health Dis.

[b33] Mair W, Dillin A (2008). Aging and survival: the genetics of life span extension by dietary restriction. Annu. Rev. Biochem.

[b34] Menni C, Kastenmuller G, Petersen AK, Bell JT, Psatha M, Tsai PC, Gieger C, Schulz H, Erte I, John S, Brosnan MJ, Wilson SG, Tsaprouni L, Lim EM, Stuckey B, Deloukas P, Mohney R, Suhre K, Spector TD, Valdes AM (2013). Metabolomic markers reveal novel pathways of ageing and early development in human populations. Int. J. Epidemiol.

[b35] Mishur RJ, Rea SL (2012). Applications of mass spectrometry to metabolomics and metabonomics: detection of biomarkers of aging and of age-related diseases. Mass Spectrom. Rev.

[b36] Muller-Linow M, Weckwerth W, Hutt MT (2007). Consistency analysis of metabolic correlation networks. BMC Syst. Biol.

[b37] Niccoli T, Partridge L (2012). Ageing as a risk factor for disease. Curr. Biol.

[b38] Palm W, Sampaio JL, Brankatschk M, Carvalho M, Mahmoud A, Shevchenko A, Eaton S (2012). Lipoproteins in *Drosophila melanogaster*–assembly, function, and influence on tissue lipid composition. PLoS Genet.

[b39] Parashar V, Rogina B (2009). dSir2 mediates the increased spontaneous physical activity in flies on calorie restriction. Aging (Albany NY).

[b40] Park LK, Friso S, Choi SW (2012). Nutritional influences on epigenetics and age-related disease. Proc. Nutr. Soc.

[b41] Pletcher SD, Macdonald SJ, Marguerie R, Certa U, Stearns SC, Goldstein DB, Partridge L (2002). Genome-wide transcript profiles in aging and calorically restricted *Drosophila melanogaster*. Curr. Biol.

[b42] Pletcher SD, Libert S, Skorupa D (2005). Flies and their golden apples: the effect of dietary restriction on *Drosophila* aging and age-dependent gene expression. Ageing Res. Rev.

[b101] Redman LM, Huffman KM, Landerman LR, Pieper CF, Bain JR, Muehlbauer MJ, Stevens RD, Wenner BR, Kraus VB, Newgard CB (2011). Effect of Caloric Restriction with and without Exercise on Metabolic Intermediates in Nonobese Men and Women. The Journal of clinical endocrinology and metabolism.

[b48] R Core Team (2013). R: A Language and Environment for Statistical Computing.

[b43] Rezzi S, Martin FP, Shanmuganayagam D, Colman RJ, Nicholson JK, Weindruch R (2009). Metabolic shifts due to long-term caloric restriction revealed in nonhuman primates. Exp. Gerontol.

[b44] Richards SE, Wang Y, Claus SP, Lawler D, Kochhar S, Holmes E, Nicholson JK (2013). Metabolic phenotype modulation by caloric restriction in a lifelong dog study. J. Proteome Res.

[b45] Sarup P, Pedersen SM, Nielsen NC, Malmendal A, Loeschcke V (2012). The metabolic profile of long-lived *Drosophila melanogaster*. PLoS ONE.

[b46] Soltow QA, Jones DP, Promislow DE (2010). A network perspective on metabolism and aging. Integr. Comp. Biol.

[b47] Southworth LK, Owen AB, Kim SK (2009). Aging mice show a decreasing correlation of gene expression within genetic modules. PLoS Genet.

[b49] Tesson BM, Breitling R, Jansen RC (2010). DiffCoEx: a simple and sensitive method to find differentially coexpressed gene modules. BMC Bioinformatics.

[b50] Tomas-Loba A, Bernardes de Jesus B, Mato JM, Blasco MA (2013). A metabolic signature predicts biological age in mice. Aging Cell.

[b51] Wang X, Wang LP, Tang H, Shan WY, Wang X, Liu D, Wu YY, Tian Q, Wang JZ, Zhu LQ (2014). Acetyl-L-carnitine rescues scopolamine-induced memory deficits by restoring insulin-like growth factor II via decreasing p53 oxidation. Neuropharmacology.

[b52] Whitaker R, Gil MP, Ding F, Tatar M, Helfand SL, Neretti N (2014). Dietary switch reveals fast coordinated gene expression changes in *Drosophila melanogaster*. Aging (Albany NY).

[b53] Williams CM, Watanabe M, Guarracino MR, Ferraro MB, Edison AS, Morgan TJ, Boroujerdi AF, Hahn DA (2014). Cold adaptation shapes the robustness of metabolic networks in *Drosophila melanogaster*. Evolution.

[b54] Young A (1997). Ageing and physiological functions. Philos. Trans. R. Soc. Lond. B Biol. Sci.

[b55] Zhang D, Sun X, Liu J, Xie X, Cui W, Zhu Y (2015). Homocysteine accelerates senescence of endothelial cells via DNA hypomethylation of human telomerase reverse transcriptase. Arterioscler. Thromb. Vasc. Biol.

[b56] Zid BM, Rogers AN, Katewa SD, Vargas MA, Kolipinski MC, Lu TA, Benzer S, Kapahi P (2009). 4E-BP extends lifespan upon dietary restriction by enhancing mitochondrial activity in *Drosophila*. Cell.

