# Supplementary figures and images for: The effects of age and dietary restriction on the tissue-specific metabolome of Drosophila
Source: Aging Cell. 2015 Jun 18;14(5):797–808. doi: 10.1111/acel.12358 (PMC4568967; doi:10.1111/acel.12358)

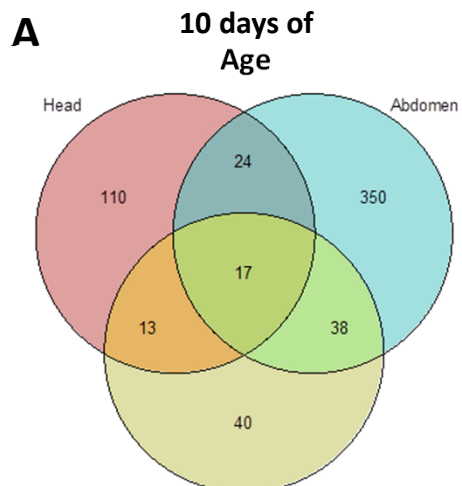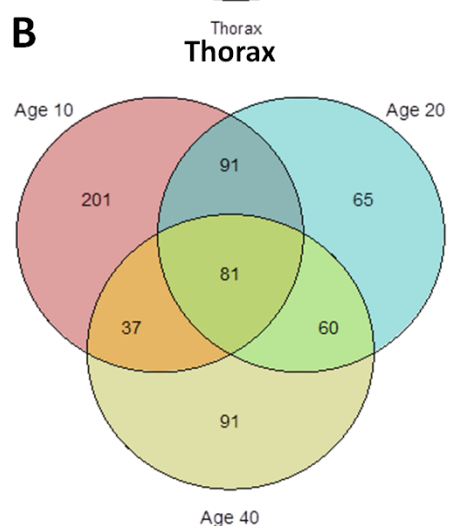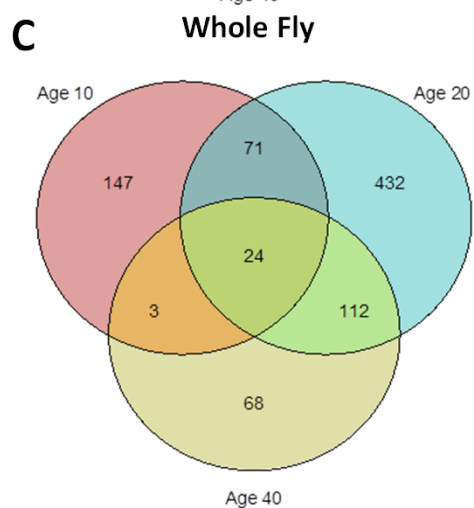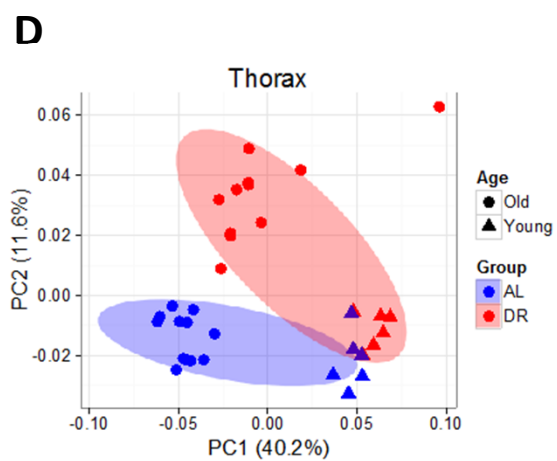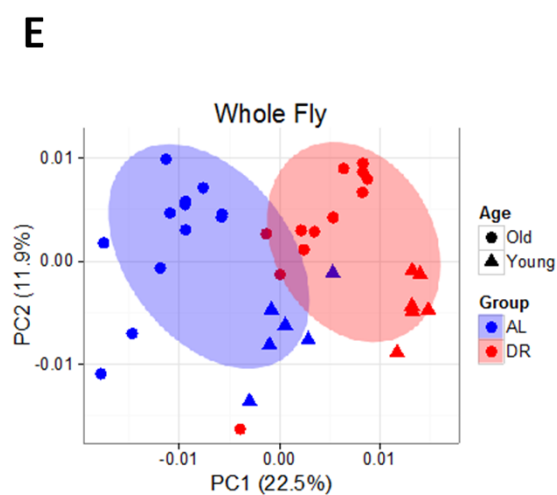

Supplement: Supplementary file 1 [file acel0014-0797-sd1.pdf]

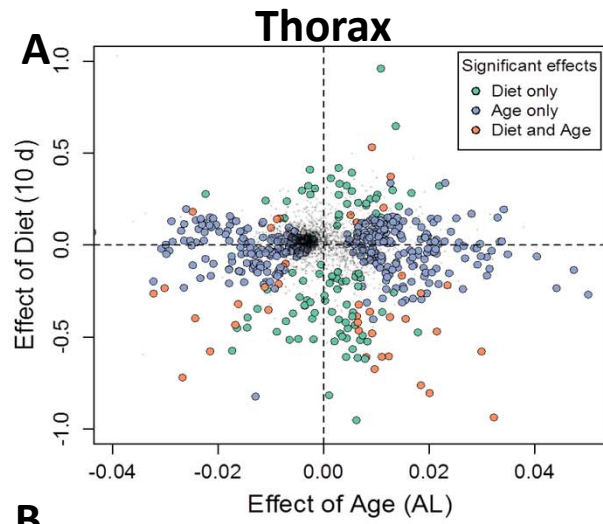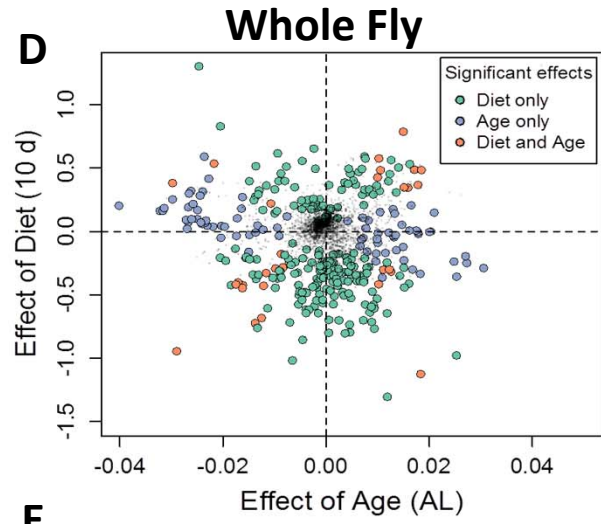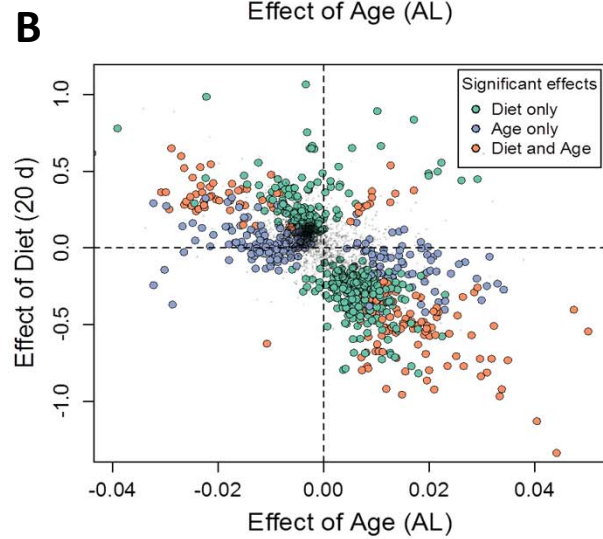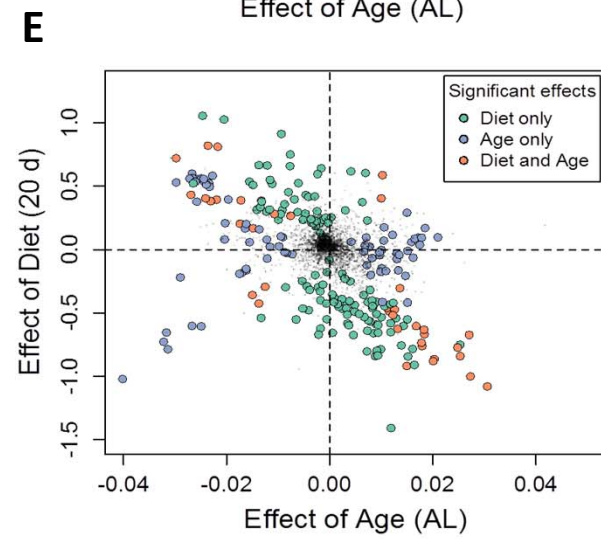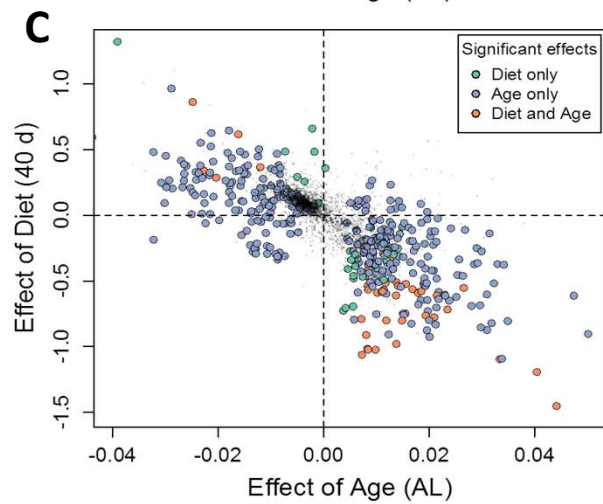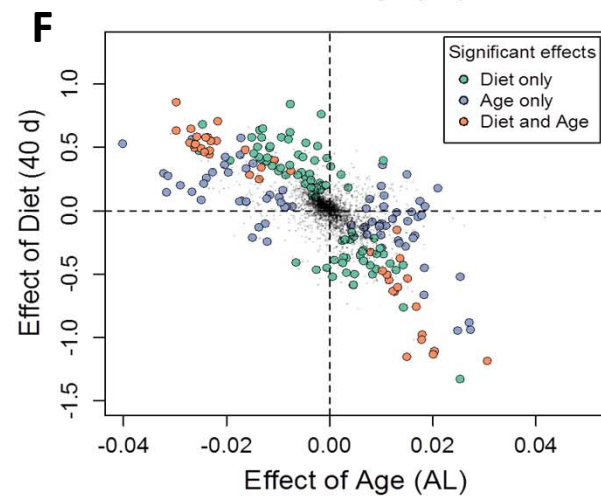

Supplement: Supplementary file 2 [file acel0014-0797-sd2.pdf]

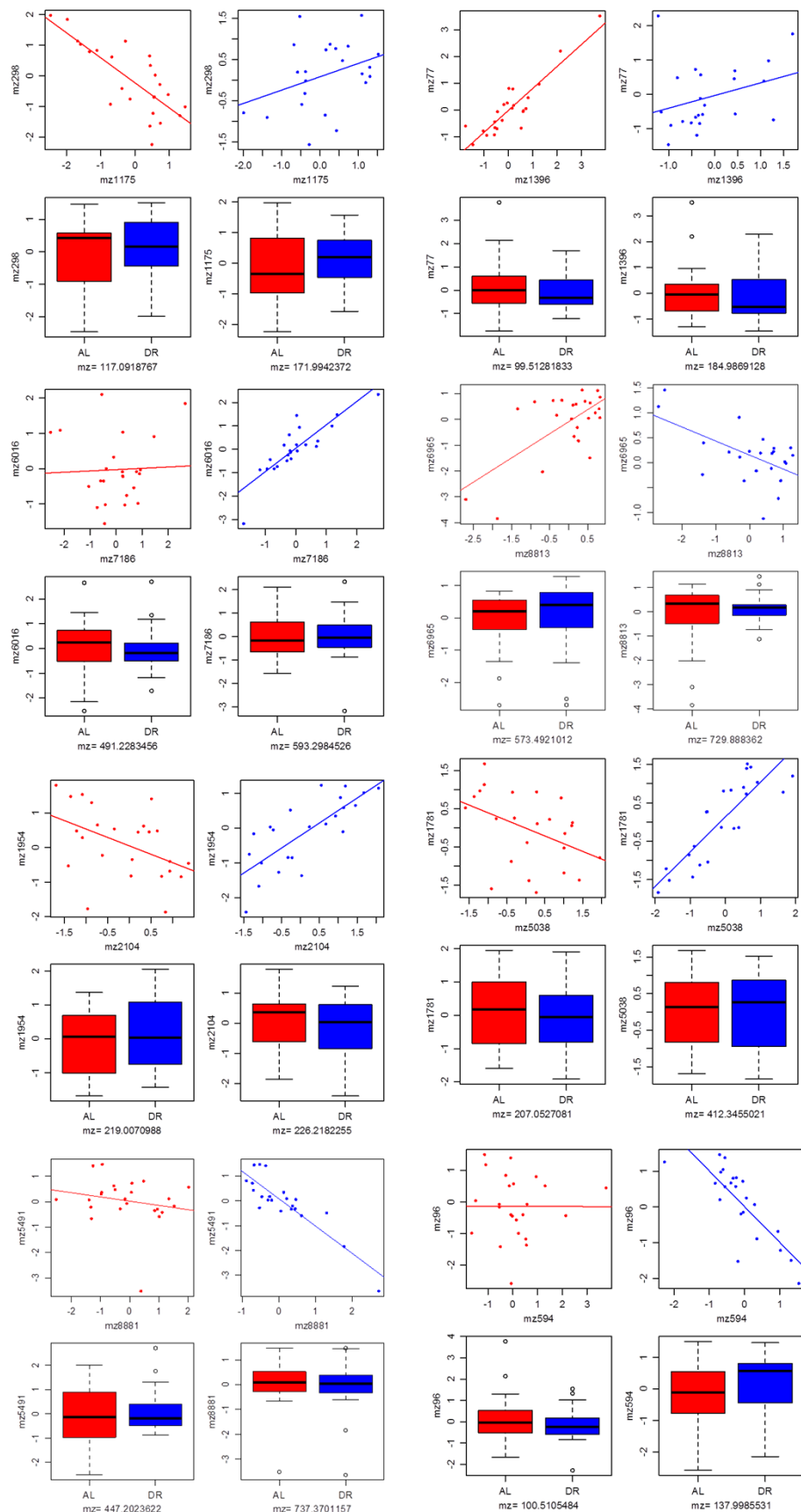

Supplement: Supplementary file 3 [file acel0014-0797-sd3.pdf]

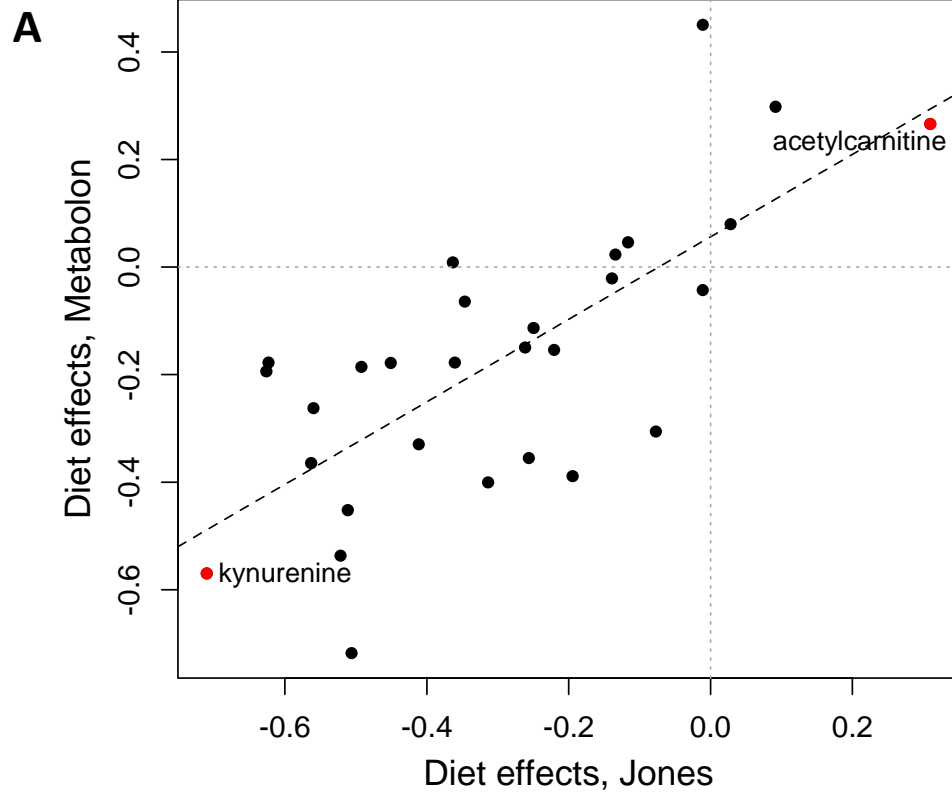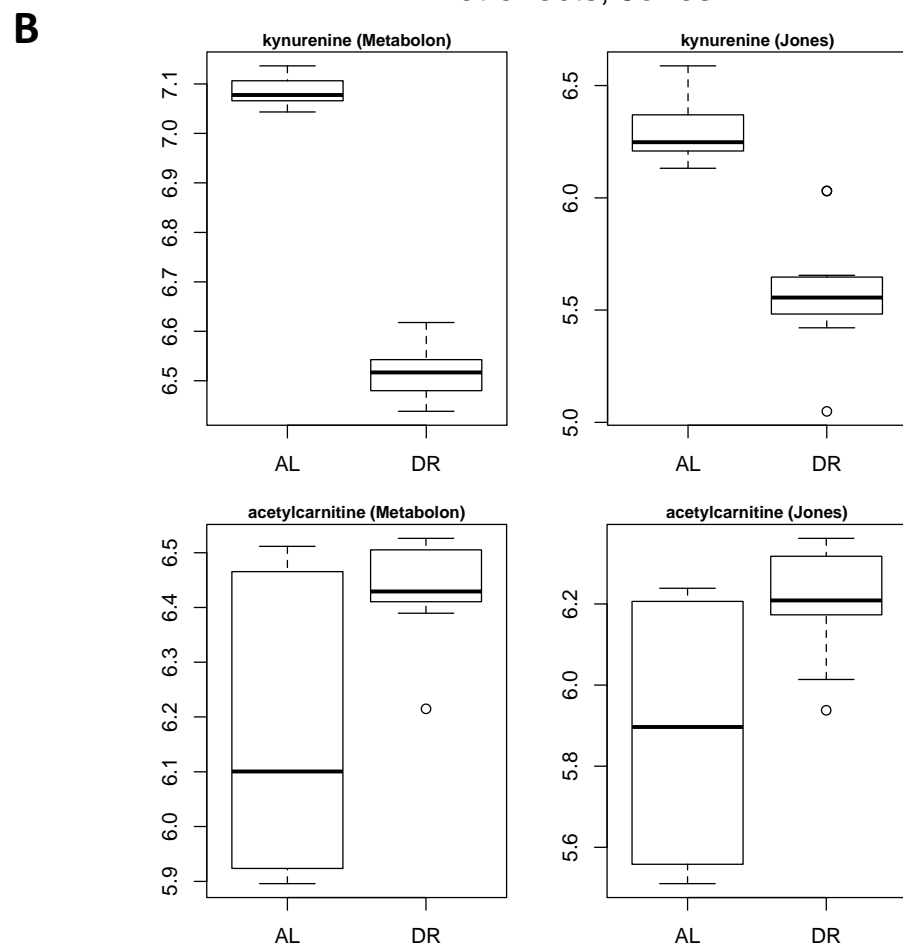

Supplement: Supplementary file 4 [file acel0014-0797-sd4.pdf]
