# Supplementary material for: The effects of age and dietary restriction on the tissue-specific metabolome of Drosophila
Source: Aging Cell. 2015 Jun 18;14(5):797–808. doi: 10.1111/acel.12358 (PMC4568967; doi:10.1111/acel.12358)
Supplement: Supplementary file 5 [file acel0014-0797-sd5.docx]

Supplemental Information

*Metabolon Data Analysis*

In addition to high resolution non-targeted metabolomics we sent samples (n = 5) from an independent experiment to Metabolon (Durham, NC) for analysis. Sample groups were sent blinded to Metabolon and only unblinded once the analysis was completed. We provide their stated methods below.

The sample preparation process was carried out using the automated MicroLab STAR® system from Hamilton Company. Recovery standards were added prior to the first step in the extraction process for quality control purposes. Sample preparation was conducted using a proprietary series of organic and aqueous extractions to remove the protein fraction while allowing maximum recovery of small molecules. The resulting extract was divided into two fractions; one for analysis by LC and one for analysis by GC. Samples were placed briefly on a TurboVap® (Zymark) to remove the organic solvent. Each sample was then frozen and dried under vacuum. Samples were then prepared for the appropriate instrument, either LC/MS or GC/MS.

For QC purposes, a number of additional samples are included with each day’s analysis. Furthermore, a selection of QC compounds is added to every sample, including those under test. These compounds are carefully chosen so as not to interfere with the measurement of the endogenous compounds. Tables 1 and 2 describe the QC samples and compounds. These QC samples are primarily used to evaluate the process control for each study as well as aiding in the data curation.

Also included were several technical replicate samples created from a homogeneous pool containing a small amount of all study samples (“Client Matrix”). Pool samples had a median relative standard deviation of 10%, while internal standards, indicating intstrument variability, had a median relative standard deviation of 4%. Both variability measures are within Metabolon’s acceptable criteria limits.

Liquid chromatography/Mass Spectrometry (LC/MS, LC/MS2): The LC/MS portion of the platform was based on a Waters ACQUITY UPLC and a Thermo-Finnigan LTQ mass spectrometer, which consisted of an electrospray ionization (ESI) source and linear ion-trap (LIT) mass analyzer. The sample extract was split into two aliquots, dried, then reconstituted in acidic or basic LC-compatible solvents, each of which contained 11 or more injection standards at fixed concentrations. One aliquot was analyzed using acidic positive ion optimized conditions and the other using basic negative ion optimized conditions in two independent injections using separate dedicated columns. Extracts reconstituted in acidic conditions were gradient eluted using water and methanol both containing 0.1% Formic acid, while the basic extracts, which also used water/methanol, contained 6.5mM Ammonium Bicarbonate. The MS analysis alternated between MS and data-dependent MS^2^ scans using dynamic exclusion.

Gas chromatography/Mass Spectrometry (GC/MS): The samples destined for GC/MS analysis were re-dried under vacuum desiccation for a minimum of 24 hours prior to being derivatized under dried nitrogen using bistrimethyl-silyl-triflouroacetamide (BSTFA). The GC column was 5% phenyl and the temperature ramp is from 40° to 300° C in a 16 minute period. Samples were analyzed on a Thermo-Finnigan Trace DSQ fast-scanning single-quadrupole mass spectrometer using electron impact ionization. The instrument was tuned and calibrated for mass resolution and mass accuracy on a daily basis. The information output from the raw data files was automatically extracted as discussed below.

Accurate Mass Determination and MS/MS fragmentation (LC/MS), (LC/MS/MS): The LC/MS portion of the platform was based on a Waters ACQUITY UPLC and a Thermo-Finnigan LTQ-FT mass spectrometer, which had a linear ion-trap (LIT) front end and a Fourier transform ion cyclotron resonance (FT-ICR) mass spectrometer backend. For ions with counts greater than 2 million, an accurate mass measurement could be performed. Accurate mass measurements could be made on the parent ion as well as fragments. The typical mass error was less than 5 ppm. Ions with less than two million counts require a greater amount of effort to characterize. Fragmentation spectra (MS/MS) were typically generated in data dependent manner, but if necessary, targeted MS/MS could be employed, such as in the case of lower level signals.

Compound identification: Compounds were identified by comparison to library entries of purified standards or recurrent unknown entities. Identification of known chemical entities was based on comparison to metabolomic library entries of purified standards. As of this writing, more than 1000 commercially available purified standard compounds had been acquired registered into LIMS for distribution to both the LC and GC platforms for determination of their analytical characteristics. The combination of chromatographic properties and mass spectra gave an indication of a match to the specific compound or an isobaric entity. Additional entities could be identified by virtue of their recurrent nature (both chromatographic and mass spectral). These compounds have the potential to be identified by future acquisition of a matching purified standard or by classical structural analysis.

Data Analysis: The dataset comprised a total of 343 compounds of known identity (named biochemicals). Following normalization to Bradford protein concentration, median scaling, imputation of missing values, if any, with the minimum observed value for each compound and log transformation median scaled data, Welch’s two-sample *t*-test was used to identify biochemicals that differed significantly between experimental groups.

**Supplemental Tables**

Table S1: Metabolic Sets Decreased on DR from Metabolites Run on the C18 column

| **Pathway** | **Head** | **Abdomen** | **Thorax10** | **Thorax20** | **Thorax40** | **Whole10** | **Whole20** | **Whole40** | **Frequency** |
| --- | --- | --- | --- | --- | --- | --- | --- | --- | --- |
| amino acids | X | X | X | X | X | X | X | X | 8 |
| tryptophan degradation to 2-amino-3-carboxymuconate semialdehyde |  | X | X | X |  | X | X |  | 5 |
| beta-alanine betaine biosynthesis | X |  |  | X |  | X | X |  | 4 |
| coenzyme A biosynthesis |  | X |  |  |  | X | X |  | 3 |
| isoleucine biosynthesis from threonine |  | X | X | X |  |  |  |  | 3 |
| dopamine degradation | X | X |  |  |  |  |  |  | 2 |
| formylTHF biosynthesis I | X | X |  |  |  |  |  |  | 2 |
| choline biosynthesis III |  | X |  |  |  | X |  |  | 2 |
| phosphatidylcholine biosynthesis |  | X |  |  |  | X |  |  | 2 |
| pyrimidine ribonucleotides interconversion |  | X |  |  |  | X |  |  | 2 |
| salvage pathways of guanine |  | X |  |  |  | X |  |  | 2 |
| salvage pathways of pyrimidine ribonucleotides |  | X |  |  |  | X |  |  | 2 |
| methionine degradation I (to homocysteine) |  |  |  | X |  |  | X |  | 2 |
| S-adenosyl-L-methionine cycle |  |  |  | X |  |  | X |  | 2 |
| folate transformations | X |  |  |  |  |  |  |  | 1 |
| isoleucine degradation I | X |  |  |  |  |  |  |  | 1 |
| leucine degradation I | X |  |  |  |  |  |  |  | 1 |
| proline degradation I | X |  |  |  |  |  |  |  | 1 |
| valine degradation I | X |  |  |  |  |  |  |  | 1 |
| asparagine biosynthesis III |  |  |  |  |  | X |  |  | 1 |
| asparaginyl-tRNA-asn biosynthesis via transamidation |  |  |  |  |  | X |  |  | 1 |
| catecholamine biosynthesis |  |  |  |  |  | X |  |  | 1 |
| de novo biosynthesis of pyrimidine deoxyribonucleotides |  |  |  |  |  | X |  |  | 1 |
| dolichyl-diphosphooligosaccharide biosynthesis |  |  |  |  |  | X |  |  | 1 |
| NAD biosynthesis from 2-amino-3-carboxymuconate semialdehyde |  |  |  |  |  | X |  |  | 1 |
| serotonin and melatonin biosynthesis |  |  |  |  |  | X |  |  | 1 |
| uridine-5'-phosphate biosynthesis |  |  |  |  |  | X |  |  | 1 |
| acyl carrier protein metabolism |  | X |  |  |  |  |  |  | 1 |
| de novo biosynthesis of pyrimidine deoxyribonucleotides |  | X |  |  |  |  |  |  | 1 |
| dolichyl-diphosphooligosaccharide biosynthesis |  | X |  |  |  |  |  |  | 1 |
| formaldehyde oxidation V (tetrahydrofolate pathway) |  | X |  |  |  |  |  |  | 1 |
| urate biosynthesis |  | X |  |  |  |  |  |  | 1 |
| citrulline-nitric oxide cycle |  |  |  | X |  |  |  |  | 1 |
| lipoate biosynthesis and incorporation II |  |  |  | X |  |  |  |  | 1 |
| purine deoxyribonucleosides degradation |  |  |  | X |  |  |  |  | 1 |
| salvage pathways of adenine |  |  |  |  |  |  | X |  | 1 |

Table S2: Metabolic Sets Enriched on DR from C18 Column

| Pathway | Head | Abdomen | Thorax10 | Thorax20 | Thorax40 | Whole10 | Whole20 | Whole40 | Frequency |
| --- | --- | --- | --- | --- | --- | --- | --- | --- | --- |
| dopamine degradation |  | X |  | X |  | X | X |  | 4 |
| GDP-glucose biosynthesis |  | X |  | X |  | X |  | X | 4 |
| glucose and glucose-1-phosphate degradation |  | X |  | X |  | X |  | X | 4 |
| glycogen degradation I |  | X |  | X |  | X |  | X | 4 |
| glycogen degradation II |  | X |  | X |  | X | X |  | 4 |
| myo-inositol biosynthesis |  | X |  | X |  | X | X |  | 4 |
| GDP-mannose biosynthesis II |  | X |  | X |  |  |  | X | 3 |
| GDP-mannose metabolism |  | X |  | X |  |  |  | X | 3 |
| glycolysis III (Thermotoga) |  | X |  | X |  | X |  |  | 3 |
| gluconeogenesis |  |  |  |  |  |  | X | X | 2 |
| glycolysis I |  |  |  | X |  |  |  | X | 2 |
| lactose degradation III |  |  |  | X |  | X |  |  | 2 |
| melibiose degradation |  |  |  | X |  | X |  |  | 2 |
| pentose phosphate pathway (non-oxidative branch) |  | X |  | X |  |  |  |  | 2 |
| pentose phosphate pathway (partial) |  | X |  | X |  |  |  |  | 2 |
| trehalose degradation II (trehalase) |  |  |  | X |  | X |  |  | 2 |
| tyrosine degradation I |  | X |  | X |  |  |  |  | 2 |
| D-mannose degradation |  |  |  |  |  |  | X |  | 1 |
| degradation of purine ribonucleosides |  | X |  |  |  |  |  |  | 1 |
| glycolysis II |  |  |  | X |  |  |  |  | 1 |
| salvage pathways of guanine |  | X |  |  |  |  |  |  | 1 |

Table S3: Metabolic path enrichment for modules in DiffCoEx

| **Column** | **Effect** | **Module** | **Pathway** | **Number Observed** | **Number in Reference** | **p-value** | **adjusted p-value** |
| --- | --- | --- | --- | --- | --- | --- | --- |
| AE | Thorax, DiffCoEx | Turquoise | Amino Acids | 7 | 20 | 0.19335 | 4.14E-03 |
| AE | Thorax, DiffCoEx | Turquoise | degradation of purine ribonucleosides | 6 | 8 | 0.00352 | 5.50E-04 |
| AE | Thorax, DiffCoEx | Turquoise | salvage pathways of adenine, hypoxanthine, and their nucleosides | 6 | 16 | 0.17219 | 3.89E-03 |
| AE | Thorax, DiffCoEx | Turquoise | NAD biosynthesis from 2-amino-3-carboxymuconate semialdehyde | 5 | 9 | 0.04346 | 1.19E-03 |
| AE | Thorax, DiffCoEx | Turquoise | gluconeogenesis | 5 | 10 | 0.07009 | 1.67E-03 |
| C18 | Thorax, SimCoEx | Brown | Amino Acids | 5 | 19 | 0.00779 | 6.04E-03 |
| AE | Thorax, SimCoEx | Blue | Amino Acids | 5 | 20 | 0.27088 | 7.40E-03 |
| AE | Thorax, DiffCoEx | Turquoise | UDP-N-acetyl-D-glucosamine biosynthesis II | 4 | 6 | 0.03398 | 1.24E-03 |
| AE | Thorax, DiffCoEx | Turquoise | isoleucine biosynthesis from threonine | 4 | 6 | 0.03398 | 1.24E-03 |
| AE | Thorax, DiffCoEx | Turquoise | arginine degradation VI (arginase 2 pathway) | 4 | 6 | 0.03398 | 1.24E-03 |
| AE | Thorax, DiffCoEx | Turquoise | arginine degradation I (arginase pathway) | 4 | 7 | 0.06436 | 1.91E-03 |
| AE | Thorax, DiffCoEx | Turquoise | salvage pathways of guanine, xanthine, and their nucleosides | 4 | 8 | 0.10472 | 3.03E-03 |
| C18 | Thorax, DiffCoEx | Turquoise | NAD biosynthesis from 2-amino-3-carboxymuconate semialdehyde | 4 | 6 | 8.00E-05 | 3.45E-03 |
| AE | Thorax, DiffCoEx | Turquoise | glycolysis I | 4 | 9 | 0.15373 | 4.89E-03 |
| AE | Thorax, DiffCoEx | Turquoise | arginine biosynthesis IV | 4 | 11 | 0.26989 | 1.23E-02 |
| AE | Thorax, DiffCoEx | Turquoise | formylTHF biosynthesis I | 4 | 12 | 0.33282 | 1.89E-02 |
| C18 | Thorax, SimCoEx | Yellow | GDP-mannose biosynthesis II | 4 | 6 | 0.00112 | 0.03464 |
| C18 | Thorax, SimCoEx | Yellow | GDP-mannose metabolism | 4 | 7 | 0.00242 | 0.03541 |
| C18 | Thorax, SimCoEx | Yellow | GDP-glucose biosynthesis | 4 | 8 | 0.00448 | 0.03641 |
| C18 | Thorax, SimCoEx | Yellow | gluconeogenesis | 4 | 10 | 0.01153 | 0.03917 |
| C18 | Thorax, SimCoEx | Green | glycogen degradation I | 4 | 10 | 0.00095 | 7.24E-02 |

Table S4: Metabolic Sets Enriched from SimEx Analysis

| **Column** | **Effect** | **Module** | **Pathway** | **Number Observed** | **Number in Reference** | **p-value** | **adjusted p-value** |
| --- | --- | --- | --- | --- | --- | --- | --- |
| AE | Thorax, DiffCoEx | Turquoise | tRNA charging pathway | 7 | 20 | 0.19335 | 4.14E-03 |
| AE | Thorax, DiffCoEx | Turquoise | degradation of purine ribonucleosides | 6 | 8 | 0.00352 | 5.50E-04 |
| AE | Thorax, DiffCoEx | Turquoise | salvage pathways of adenine, hypoxanthine, and their nucleosides | 6 | 16 | 0.17219 | 3.89E-03 |
| AE | Thorax, DiffCoEx | Turquoise | NAD biosynthesis from 2-amino-3-carboxymuconate semialdehyde | 5 | 9 | 0.04346 | 1.19E-03 |
| AE | Thorax, DiffCoEx | Turquoise | gluconeogenesis | 5 | 10 | 0.07009 | 1.67E-03 |
| C18 | Thorax, SimCoEx | Brown | tRNA charging pathway | 5 | 19 | 0.00779 | 6.04E-03 |
| AE | Thorax, SimCoEx | Blue | tRNA charging pathway | 5 | 20 | 0.27088 | 7.40E-03 |
| AE | Thorax, DiffCoEx | Turquoise | UDP-N-acetyl-D-glucosamine biosynthesis II | 4 | 6 | 0.03398 | 1.24E-03 |
| AE | Thorax, DiffCoEx | Turquoise | isoleucine biosynthesis from threonine | 4 | 6 | 0.03398 | 1.24E-03 |
| AE | Thorax, DiffCoEx | Turquoise | arginine degradation VI (arginase 2 pathway) | 4 | 6 | 0.03398 | 1.24E-03 |
| AE | Thorax, DiffCoEx | Turquoise | arginine degradation I (arginase pathway) | 4 | 7 | 0.06436 | 1.91E-03 |
| AE | Thorax, DiffCoEx | Turquoise | salvage pathways of guanine, xanthine, and their nucleosides | 4 | 8 | 0.10472 | 3.03E-03 |
| C18 | Thorax, DiffCoEx | Turquoise | NAD biosynthesis from 2-amino-3-carboxymuconate semialdehyde | 4 | 6 | 8.00E-05 | 3.45E-03 |
| AE | Thorax, DiffCoEx | Turquoise | glycolysis I | 4 | 9 | 0.15373 | 4.89E-03 |
| AE | Thorax, DiffCoEx | Turquoise | arginine biosynthesis IV | 4 | 11 | 0.26989 | 1.23E-02 |
| AE | Thorax, DiffCoEx | Turquoise | formylTHF biosynthesis I | 4 | 12 | 0.33282 | 1.89E-02 |
| C18 | Thorax, SimCoEx | Yellow | GDP-mannose biosynthesis II | 4 | 6 | 0.00112 | 0.03464 |
| C18 | Thorax, SimCoEx | Yellow | GDP-mannose metabolism | 4 | 7 | 0.00242 | 0.03541 |
| C18 | Thorax, SimCoEx | Yellow | GDP-glucose biosynthesis | 4 | 8 | 0.00448 | 0.03641 |
| C18 | Thorax, SimCoEx | Yellow | gluconeogenesis | 4 | 10 | 0.01153 | 0.03917 |
| C18 | Thorax, SimCoEx | Green | glycogen degradation I | 4 | 10 | 0.00095 | 7.24E-02 |
| AE | Thorax, DiffCoEx | Blue | uridine-5'-phosphate biosynthesis | 3 | 9 | 0.017886583 | 4.77E-04 |
| AE | Thorax, DiffCoEx | Blue | salvage pathways of pyrimidine ribonucleotides | 3 | 9 | 0.017886583 | 4.77E-04 |
| AE | Thorax, SimCoEx | Turquoise | 4-hydroxybenzoate biosynthesis I (eukaryotes) | 3 | 6 | 0.00559 | 5.50E-04 |
| AE | Thorax, DiffCoEx | Turquoise | glutamate biosynthesis I | 3 | 3 | 0.01437 | 1.17E-03 |
| AE | Thorax, DiffCoEx | Turquoise | glutamine degradation II | 3 | 3 | 0.01437 | 1.17E-03 |
| AE | Thorax, SimCoEx | Blue | glutathione redox reactions II | 3 | 3 | 0.00544 | 1.37E-03 |
| AE | Thorax, SimCoEx | Blue | glutathione redox reactions I | 3 | 3 | 0.00544 | 1.37E-03 |
| AE | Thorax, SimCoEx | Blue | glutamate biosynthesis I | 3 | 3 | 0.00544 | 1.37E-03 |
| AE | Thorax, SimCoEx | Blue | glutamine degradation II | 3 | 3 | 0.00544 | 1.37E-03 |
| AE | Thorax, SimCoEx | Turquoise | salvage pathways of adenine, hypoxanthine, and their nucleosides | 3 | 16 | 0.09524 | 1.56E-03 |
| AE | Thorax, DiffCoEx | Turquoise | choline biosynthesis III | 3 | 4 | 0.0471 | 2.17E-03 |
| AE | Thorax, DiffCoEx | Turquoise | degradation of pyrimidine deoxyribonucleosides | 3 | 4 | 0.0471 | 2.17E-03 |
| AE | Thorax, SimCoEx | Blue | pyrimidine ribonucleotides interconversion | 3 | 6 | 0.07189 | 3.18E-03 |
| C18 | Thorax, DiffCoEx | Turquoise | 5-aminoimidazole ribonucleotide biosynthesis I | 3 | 5 | 0.00113 | 3.67E-03 |
| C18 | Thorax, DiffCoEx | Turquoise | 4-hydroxyproline degradation I | 3 | 6 | 0.00218 | 3.79E-03 |
| AE | Thorax, DiffCoEx | Turquoise | glutaminyl-tRNAgln biosynthesis via transamidation | 3 | 5 | 0.09677 | 4.12E-03 |
| AE | Thorax, DiffCoEx | Turquoise | oxidative ethanol degradation III | 3 | 5 | 0.09677 | 4.12E-03 |
| AE | Thorax, DiffCoEx | Turquoise | oxidative ethanol degradation I | 3 | 5 | 0.09677 | 4.12E-03 |
| AE | Thorax, DiffCoEx | Turquoise | proline degradation I | 3 | 5 | 0.09677 | 4.12E-03 |
| AE | Thorax, DiffCoEx | Turquoise | 4-hydroxyproline degradation I | 3 | 5 | 0.09677 | 4.12E-03 |
| AE | Thorax, DiffCoEx | Turquoise | pentose phosphate pathway (oxidative branch) | 3 | 5 | 0.09677 | 4.12E-03 |
| AE | Thorax, SimCoEx | Blue | proline biosynthesis II | 3 | 7 | 0.1099 | 4.30E-03 |
| AE | Thorax, SimCoEx | Blue | proline biosynthesis III | 3 | 7 | 0.1099 | 4.30E-03 |
| C18 | Thorax, DiffCoEx | Turquoise | formylTHF biosynthesis I | 3 | 13 | 0.02438 | 5.23E-03 |
| AE | Thorax, SimCoEx | Red | isoleucine biosynthesis from threonine | 3 | 6 | 0.00071 | 5.52E-03 |
| AE | Thorax, SimCoEx | Blue | salvage pathways of guanine, xanthine, and their nucleosides | 3 | 8 | 0.15384 | 5.81E-03 |
| AE | Thorax, SimCoEx | Blue | degradation of purine ribonucleosides | 3 | 8 | 0.15384 | 5.81E-03 |
| C18 | Thorax, SimCoEx | Brown | tyrosine degradation I | 3 | 6 | 0.00571 | 6.62E-03 |
| AE | Thorax, DiffCoEx | Brown | tRNA charging pathway | 3 | 20 | 0.174872581 | 6.87E-03 |
| C18 | Thorax, SimCoEx | Brown | arginine degradation VI (arginase 2 pathway) | 3 | 7 | 0.00951 | 7.17E-03 |
| AE | Thorax, DiffCoEx | Turquoise | phosphatidylcholine biosynthesis | 3 | 6 | 0.15956 | 7.64E-03 |
| AE | Thorax, DiffCoEx | Turquoise | L-carnitine biosynthesis | 3 | 6 | 0.15956 | 7.64E-03 |
| AE | Thorax, DiffCoEx | Turquoise | glutathione biosynthesis | 3 | 6 | 0.15956 | 7.64E-03 |
| AE | Thorax, DiffCoEx | Turquoise | serine biosynthesis | 3 | 6 | 0.15956 | 7.64E-03 |
| AE | Thorax, DiffCoEx | Turquoise | asparagine biosynthesis III | 3 | 6 | 0.15956 | 7.64E-03 |
| AE | Thorax, DiffCoEx | Turquoise | asparaginyl-tRNAasn biosynthesis via transamidation | 3 | 6 | 0.15956 | 7.64E-03 |
| AE | Thorax, DiffCoEx | Turquoise | urate biosynthesis | 3 | 6 | 0.15956 | 7.64E-03 |
| AE | Thorax, DiffCoEx | Turquoise | isoleucine degradation I | 3 | 6 | 0.15956 | 7.64E-03 |
| AE | Thorax, SimCoEx | Blue | CMP-N-acetylneuraminate biosynthesis I (eukaryotes) | 3 | 9 | 0.2022 | 7.75E-03 |
| AE | Thorax, SimCoEx | Blue | NAD biosynthesis from 2-amino-3-carboxymuconate semialdehyde | 3 | 9 | 0.2022 | 7.75E-03 |
| -- | DR, DiffCoEx | Black | asparagine biosynthesis III | 3 | 6 | 0.0023 | 1.00E-02 |
| AE | Thorax, SimCoEx | Blue | 5-aminoimidazole ribonucleotide biosynthesis I | 3 | 10 | 0.25349 | 1.02E-02 |
| AE | Thorax, DiffCoEx | Turquoise | proline biosynthesis II | 3 | 7 | 0.23096 | 1.36E-02 |
| AE | Thorax, DiffCoEx | Turquoise | proline biosynthesis III | 3 | 7 | 0.23096 | 1.36E-02 |
| AE | Thorax, DiffCoEx | Turquoise | purine deoxyribonucleosides degradation | 3 | 7 | 0.23096 | 1.36E-02 |
| AE | Thorax, DiffCoEx | Turquoise | citrulline-nitric oxide cycle | 3 | 7 | 0.23096 | 1.36E-02 |
| -- | DR, DiffCoEx | Black | tRNA charging pathway | 3 | 20 | 0.07949 | 1.72E-02 |
| AE | Thorax, DiffCoEx | Turquoise | tryptophan degradation to 2-amino-3-carboxymuconate semialdehyde | 3 | 8 | 0.3067 | 2.28E-02 |
| AE | Thorax, DiffCoEx | Turquoise | leucine degradation I | 3 | 8 | 0.3067 | 2.28E-02 |
| AE | Thorax, DiffCoEx | Turquoise | glycolysis II | 3 | 8 | 0.3067 | 2.28E-02 |
| AE | Thorax, DiffCoEx | Turquoise | glycolysis III (Thermotoga) | 3 | 8 | 0.3067 | 2.28E-02 |
| C18 | Thorax, DiffCoEx | Cyan | glucose and glucose-1-phosphate degradation | 3 | 7 | 0.01298 | 3.39E-02 |
| AE | Thorax, DiffCoEx | Turquoise | salvage pathways of pyrimidine ribonucleotides | 3 | 9 | 0.38318 | 3.63E-02 |
| C18 | Thorax, SimCoEx | Pink | dopamine degradation | 3 | 9 | 0.00195 | 3.94E-02 |
| C18 | Thorax, SimCoEx | Yellow | glucose and glucose-1-phosphate degradation | 3 | 7 | 0.02412 | 0.04758 |
| AE | Thorax, SimCoEx | Magenta | GDP-mannose biosynthesis II | 3 | 5 | 0.00334 | 5.18E-02 |
| AE | Thorax, SimCoEx | Pink | GDP-mannose biosynthesis II | 3 | 5 | 0.00334 | 5.18E-02 |
| AE | Thorax, SimCoEx | Magenta | GDP-mannose metabolism | 3 | 6 | 0.00633 | 5.43E-02 |
| AE | Thorax, SimCoEx | Magenta | GDP-glucose biosynthesis | 3 | 6 | 0.00633 | 5.43E-02 |
| AE | Thorax, SimCoEx | Magenta | glucose and glucose-1-phosphate degradation | 3 | 6 | 0.00633 | 5.43E-02 |
| AE | Thorax, SimCoEx | Pink | GDP-mannose metabolism | 3 | 6 | 0.00633 | 5.43E-02 |
| AE | Thorax, SimCoEx | Pink | GDP-glucose biosynthesis | 3 | 6 | 0.00633 | 5.43E-02 |
| AE | Thorax, SimCoEx | Pink | glucose and glucose-1-phosphate degradation | 3 | 6 | 0.00633 | 5.43E-02 |
| AE | Thorax, SimCoEx | Magenta | glycogen degradation I | 3 | 7 | 0.01051 | 5.73E-02 |
| AE | Thorax, SimCoEx | Pink | glycogen degradation I | 3 | 7 | 0.01051 | 5.73E-02 |
| -- | AL, DiffCoEx | Red | acyl carrier protein metabolism | 2 | 3 | 0.00188 | 0.00E+00 |
| -- | AL, DiffCoEx | Red | dopamine degradation | 2 | 11 | 0.03041 | 0.00E+00 |
| AE | Thorax, DiffCoEx | Green | inosine-5'-phosphate biosynthesis II | 2 | 7 | 0.015363531 | 4.47E-04 |
| AE | Thorax, DiffCoEx | Green | 5-aminoimidazole ribonucleotide biosynthesis I | 2 | 10 | 0.031248318 | 8.22E-04 |
| AE | Thorax, SimCoEx | Turquoise | sorbitol utilization | 2 | 3 | 0.01385 | 9.70E-04 |
| AE | Thorax, SimCoEx | Turquoise | choline biosynthesis III | 2 | 4 | 0.02645 | 1.23E-03 |
| AE | Thorax, SimCoEx | Turquoise | fatty acid β-oxidation II (core pathway) | 2 | 4 | 0.02645 | 1.23E-03 |
| AE | Thorax, SimCoEx | Turquoise | fatty acid β-oxidation I | 2 | 4 | 0.02645 | 1.23E-03 |
| AE | Thorax, SimCoEx | Turquoise | aerobic respiration -- electron donor II | 2 | 4 | 0.02645 | 1.23E-03 |
| AE | Thorax, SimCoEx | Turquoise | phosphatidylethanolamine biosynthesis II | 2 | 5 | 0.04213 | 1.54E-03 |
| AE | Thorax, SimCoEx | Turquoise | oxidative ethanol degradation III | 2 | 5 | 0.04213 | 1.54E-03 |
| AE | Thorax, SimCoEx | Turquoise | oxidative ethanol degradation I | 2 | 5 | 0.04213 | 1.54E-03 |
| AE | Thorax, SimCoEx | Turquoise | proline degradation I | 2 | 5 | 0.04213 | 1.54E-03 |
| AE | Thorax, SimCoEx | Turquoise | tyrosine degradation I | 2 | 5 | 0.04213 | 1.54E-03 |
| -- | AL, DiffCoEx | Blue | pyruvate fermentation to lactate | 2 | 3 | 0.00235 | 1.55E-03 |
| -- | AL, DiffCoEx | Turquoise | salvage pathways of pyrimidine ribonucleotides | 2 | 9 | 0.03079 | 1.67E-03 |
| -- | AL, DiffCoEx | Turquoise | de novo biosynthesis of pyrimidine deoxyribonucleotides | 2 | 9 | 0.03079 | 1.67E-03 |
| AE | Thorax, DiffCoEx | Brown | heme degradation | 2 | 2 | 0.005266475 | 1.67E-03 |
| AE | Thorax, SimCoEx | Turquoise | phosphatidylcholine biosynthesis | 2 | 6 | 0.06039 | 1.91E-03 |
| AE | Thorax, SimCoEx | Turquoise | glutathione biosynthesis | 2 | 6 | 0.06039 | 1.91E-03 |
| AE | Thorax, SimCoEx | Turquoise | arginine degradation VI (arginase 2 pathway) | 2 | 6 | 0.06039 | 1.91E-03 |
| AE | Thorax, SimCoEx | Turquoise | aerobic respiration -- electron donors reaction list | 2 | 6 | 0.06039 | 1.91E-03 |
| AE | Thorax, DiffCoEx | Blue | pentose phosphate pathway (partial) | 2 | 6 | 0.055665861 | 2.12E-03 |
| AE | Thorax, DiffCoEx | Blue | pentose phosphate pathway (non-oxidative branch) | 2 | 6 | 0.055665861 | 2.12E-03 |
| -- | AL, DiffCoEx | Blue | aerobic respiration -- electron donors reaction list | 2 | 6 | 0.01117 | 2.18E-03 |
| AE | Thorax, SimCoEx | Turquoise | purine deoxyribonucleosides degradation | 2 | 7 | 0.08083 | 2.34E-03 |
| AE | Thorax, SimCoEx | Turquoise | arginine degradation I (arginase pathway) | 2 | 7 | 0.08083 | 2.34E-03 |
| AE | Thorax, SimCoEx | Turquoise | citrulline-nitric oxide cycle | 2 | 7 | 0.08083 | 2.34E-03 |
| -- | AL, DiffCoEx | Blue | purine deoxyribonucleosides degradation | 2 | 7 | 0.01536 | 2.43E-03 |
| AE | Thorax, SimCoEx | Turquoise | glycolysis II | 2 | 8 | 0.10305 | 2.84E-03 |
| AE | Thorax, SimCoEx | Turquoise | TCA cycle variation III (eukaryotic) | 2 | 8 | 0.10305 | 2.84E-03 |
| AE | Thorax, DiffCoEx | Brown | arginine degradation X (arginine monooxygenase pathway) | 2 | 4 | 0.02870773 | 2.96E-03 |
| AE | Thorax, SimCoEx | Turquoise | NAD biosynthesis from 2-amino-3-carboxymuconate semialdehyde | 2 | 9 | 0.12671 | 3.40E-03 |
| AE | Thorax, SimCoEx | Turquoise | salvage pathways of pyrimidine ribonucleotides | 2 | 9 | 0.12671 | 3.40E-03 |
| AE | Thorax, SimCoEx | Turquoise | glycolysis I | 2 | 9 | 0.12671 | 3.40E-03 |
| AE | Thorax, SimCoEx | Blue | glutamate biosynthesis III | 2 | 2 | 0.03114 | 3.70E-03 |
| AE | Thorax, SimCoEx | Blue | glutathione-mediated detoxification | 2 | 2 | 0.03114 | 3.70E-03 |
| AE | Thorax, SimCoEx | Blue | glutamine degradation I | 2 | 2 | 0.03114 | 3.70E-03 |
| AE | Thorax, DiffCoEx | Blue | salvage pathways of guanine, xanthine, and their nucleosides | 2 | 8 | 0.09537 | 3.80E-03 |
| AE | Thorax, DiffCoEx | Blue | glycolysis II | 2 | 8 | 0.095366584 | 3.80E-03 |
| AE | Thorax, DiffCoEx | Blue | glycolysis III (Thermotoga) | 2 | 8 | 0.095366584 | 3.80E-03 |
| AE | Thorax, SimCoEx | Turquoise | gluconeogenesis | 2 | 10 | 0.15153 | 4.04E-03 |
| AE | Thorax, SimCoEx | Turquoise | 5-aminoimidazole ribonucleotide biosynthesis I | 2 | 10 | 0.15153 | 4.04E-03 |
| -- | DR, DiffCoEx | Turquoise | paraoxon degradation | 2 | 2 | 0.00346 | 4.05E-03 |
| C18 | Thorax, DiffCoEx | Turquoise | glutamine degradation I | 2 | 2 | 0.00253 | 4.67E-03 |
| AE | Thorax, SimCoEx | Turquoise | arginine biosynthesis IV | 2 | 11 | 0.17722 | 4.76E-03 |
| AE | Thorax, SimCoEx | Turquoise | dopamine degradation | 2 | 11 | 0.17722 | 4.76E-03 |
| AE | Thorax, DiffCoEx | Brown | isoleucine biosynthesis from threonine | 2 | 6 | 0.065268719 | 4.92E-03 |
| AE | Thorax, DiffCoEx | Brown | arginine degradation VI (arginase 2 pathway) | 2 | 6 | 0.065268719 | 4.92E-03 |
| AE | Thorax, DiffCoEx | Blue | de novo biosynthesis of pyrimidine deoxyribonucleotides | 2 | 9 | 0.117503727 | 4.97E-03 |
| AE | Thorax, DiffCoEx | Blue | glycolysis I | 2 | 9 | 0.117503727 | 4.97E-03 |
| AE | Thorax, SimCoEx | Brown | salvage pathways of pyrimidine ribonucleotides | 2 | 9 | 0.00867 | 5.08E-03 |
| C18 | Thorax, DiffCoEx | Turquoise | UDP-N-acetyl-D-glucosamine biosynthesis II | 2 | 3 | 0.00734 | 5.40E-03 |
| C18 | Thorax, DiffCoEx | Turquoise | glutaryl-CoA degradation | 2 | 3 | 0.00734 | 5.40E-03 |
| -- | AL, DiffCoEx | Blue | salvage pathways of adenine, hypoxanthine, and their nucleosides | 2 | 16 | 0.07512 | 5.85E-03 |
| C18 | Thorax, DiffCoEx | Turquoise | glutamine biosynthesis I | 2 | 4 | 0.01422 | 6.22E-03 |
| C18 | Thorax, DiffCoEx | Turquoise | glycine cleavage complex | 2 | 4 | 0.01422 | 6.22E-03 |
| AE | Thorax, SimCoEx | Blue | proline degradation II | 2 | 3 | 0.08255 | 6.25E-03 |
| AE | Thorax, SimCoEx | Blue | formaldehyde oxidation II (glutathione-dependent) | 2 | 3 | 0.08255 | 6.25E-03 |
| AE | Thorax, DiffCoEx | Blue | gluconeogenesis | 2 | 10 | 0.140788849 | 6.40E-03 |
| AE | Thorax, DiffCoEx | Turquoise | glutamate biosynthesis III | 2 | 2 | 0.05939 | 6.62E-03 |
| AE | Thorax, DiffCoEx | Turquoise | glutathione-mediated detoxification | 2 | 2 | 0.05939 | 6.62E-03 |
| AE | Thorax, DiffCoEx | Turquoise | glutamine degradation I | 2 | 2 | 0.05939 | 6.62E-03 |
| -- | AL, DiffCoEx | Brown | 4-hydroxyproline degradation I | 2 | 5 | 0.01409 | 6.85E-03 |
| C18 | Thorax, DiffCoEx | Turquoise | glutamate biosynthesis I | 2 | 5 | 0.02294 | 7.12E-03 |
| C18 | Thorax, DiffCoEx | Turquoise | glutaminyl-tRNAgln biosynthesis via transamidation | 2 | 5 | 0.02294 | 7.12E-03 |
| C18 | Thorax, DiffCoEx | Turquoise | aspartate degradation II | 2 | 5 | 0.02294 | 7.12E-03 |
| C18 | Thorax, DiffCoEx | Turquoise | proline degradation I | 2 | 5 | 0.02294 | 7.12E-03 |
| C18 | Thorax, DiffCoEx | Turquoise | glutamine degradation II | 2 | 5 | 0.02294 | 7.12E-03 |
| -- | AL, DiffCoEx | Brown | L-carnitine biosynthesis | 2 | 6 | 0.02062 | 7.57E-03 |
| AE | Thorax, SimCoEx | Red | 4-hydroxyproline degradation I | 2 | 5 | 0.01107 | 7.58E-03 |
| AE | Thorax, DiffCoEx | Yellow | purine deoxyribonucleosides degradation | 2 | 7 | 0.08398 | 7.76E-03 |
| AE | Thorax, SimCoEx | Red | isoleucine degradation I | 2 | 6 | 0.01625 | 8.09E-03 |
| C18 | Thorax, DiffCoEx | Turquoise | folate polyglutamylation I | 2 | 6 | 0.03332 | 8.10E-03 |
| C18 | Thorax, DiffCoEx | Turquoise | serine biosynthesis | 2 | 6 | 0.03332 | 8.10E-03 |
| C18 | Thorax, DiffCoEx | Turquoise | asparagine biosynthesis III | 2 | 6 | 0.03332 | 8.10E-03 |
| C18 | Thorax, DiffCoEx | Turquoise | asparaginyl-tRNAasn biosynthesis via transamidation | 2 | 6 | 0.03332 | 8.10E-03 |
| C18 | Thorax, DiffCoEx | Turquoise | valine degradation I | 2 | 6 | 0.03332 | 8.10E-03 |
| C18 | Thorax, DiffCoEx | Turquoise | isoleucine degradation I | 2 | 6 | 0.03332 | 8.10E-03 |
| -- | AL, DiffCoEx | Brown | purine deoxyribonucleosides degradation | 2 | 7 | 0.02816 | 8.36E-03 |
| C18 | Thorax, SimCoEx | Brown | glutamate dependent acid resistance | 2 | 2 | 0.00491 | 8.95E-03 |
| AE | Thorax, SimCoEx | Red | leucine degradation I | 2 | 8 | 0.02906 | 9.17E-03 |
| AE | Thorax, SimCoEx | Blue | glutamine biosynthesis I | 2 | 4 | 0.14618 | 9.82E-03 |
| AE | Thorax, SimCoEx | Blue | mevalonate pathway | 2 | 4 | 0.14618 | 9.82E-03 |
| AE | Thorax, DiffCoEx | Yellow | degradation of purine ribonucleosides | 2 | 8 | 0.10696 | 9.87E-03 |
| C18 | Thorax, DiffCoEx | Turquoise | uridine-5'-phosphate biosynthesis | 2 | 8 | 0.05834 | 1.04E-02 |
| C18 | Thorax, DiffCoEx | Turquoise | pyrimidine ribonucleotides interconversion | 2 | 8 | 0.05834 | 1.04E-02 |
| C18 | Thorax, DiffCoEx | Turquoise | arginine degradation I (arginase pathway) | 2 | 8 | 0.05834 | 1.04E-02 |
| C18 | Thorax, SimCoEx | Brown | proline degradation II | 2 | 3 | 0.01406 | 1.14E-02 |
| C18 | Thorax, SimCoEx | Brown | arginine degradation X (arginine monooxygenase pathway) | 2 | 3 | 0.01406 | 1.14E-02 |
| -- | DR, DiffCoEx | Black | aspartate biosynthesis | 2 | 2 | 0.00262 | 1.16E-02 |
| -- | DR, DiffCoEx | Black | asparagine degradation I | 2 | 2 | 0.00262 | 1.16E-02 |
| C18 | Thorax, DiffCoEx | Turquoise | leucine degradation I | 2 | 9 | 0.07266 | 1.16E-02 |
| -- | DR, DiffCoEx | Black | aspartate degradation II | 2 | 4 | 0.01471 | 1.42E-02 |
| C18 | Thorax, SimCoEx | Brown | 4-hydroxyphenylpyruvate biosynthesis | 2 | 4 | 0.02686 | 1.44E-02 |
| C18 | Thorax, SimCoEx | Brown | 2-methylbutyrate biosynthesis | 2 | 4 | 0.02686 | 1.44E-02 |
| C18 | Thorax, DiffCoEx | Turquoise | folate transformations | 2 | 11 | 0.10418 | 1.45E-02 |
| AE | Thorax, SimCoEx | Blue | glutaminyl-tRNAgln biosynthesis via transamidation | 2 | 5 | 0.21617 | 1.45E-02 |
| AE | Thorax, SimCoEx | Blue | proline degradation I | 2 | 5 | 0.21617 | 1.45E-02 |
| AE | Thorax, SimCoEx | Blue | valine degradation I | 2 | 5 | 0.21617 | 1.45E-02 |
| AE | Thorax, SimCoEx | Turquoise | tRNA charging pathway | 2 | 20 | 0.41665 | 1.51E-02 |
| AE | Thorax, DiffCoEx | Turquoise | fatty acids biosynthesis (yeast) | 2 | 3 | 0.14942 | 1.64E-02 |
| AE | Thorax, DiffCoEx | Turquoise | glutathione redox reactions II | 2 | 3 | 0.14942 | 1.64E-02 |
| AE | Thorax, DiffCoEx | Turquoise | glutathione redox reactions I | 2 | 3 | 0.14942 | 1.64E-02 |
| AE | Thorax, DiffCoEx | Turquoise | methylglyoxal degradation I | 2 | 3 | 0.14942 | 1.64E-02 |
| AE | Thorax, DiffCoEx | Turquoise | acetate conversion to acetyl-CoA | 2 | 3 | 0.14942 | 1.64E-02 |
| AE | Thorax, DiffCoEx | Turquoise | proline degradation II | 2 | 3 | 0.14942 | 1.64E-02 |
| AE | Thorax, DiffCoEx | Turquoise | N-acetylglucosamine degradation | 2 | 3 | 0.14942 | 1.64E-02 |
| AE | Thorax, DiffCoEx | Turquoise | sorbitol utilization | 2 | 3 | 0.14942 | 1.64E-02 |
| AE | Thorax, DiffCoEx | Turquoise | formaldehyde oxidation II (glutathione-dependent) | 2 | 3 | 0.14942 | 1.64E-02 |
| -- | DR, DiffCoEx | Turquoise | salvage pathways of pyrimidine ribonucleotides | 2 | 9 | 0.09542 | 1.67E-02 |
| -- | DR, DiffCoEx | Black | folate polyglutamylation I | 2 | 6 | 0.03442 | 1.72E-02 |
| -- | DR, DiffCoEx | Black | asparaginyl-tRNAasn biosynthesis via transamidation | 2 | 6 | 0.03442 | 1.72E-02 |
| AE | Thorax, SimCoEx | Red | tRNA charging pathway | 2 | 20 | 0.15282 | 1.75E-02 |
| C18 | Thorax, SimCoEx | Brown | proline degradation I | 2 | 5 | 0.04277 | 1.78E-02 |
| C18 | Thorax, SimCoEx | Brown | β-alanine betaine biosynthesis | 2 | 5 | 0.04277 | 1.78E-02 |
| -- | AL, DiffCoEx | Brown | salvage pathways of adenine, hypoxanthine, and their nucleosides | 2 | 16 | 0.12929 | 1.80E-02 |
| C18 | Thorax, DiffCoEx | Yellow | coenzyme A biosynthesis | 2 | 7 | 0.00838 | 2.01E-02 |
| AE | Thorax, SimCoEx | Blue | glutathione biosynthesis | 2 | 6 | 0.2883 | 2.04E-02 |
| AE | Thorax, SimCoEx | Blue | UDP-N-acetyl-D-glucosamine biosynthesis II | 2 | 6 | 0.2883 | 2.04E-02 |
| AE | Thorax, SimCoEx | Blue | isoleucine biosynthesis from threonine | 2 | 6 | 0.2883 | 2.04E-02 |
| AE | Thorax, SimCoEx | Blue | asparagine biosynthesis III | 2 | 6 | 0.2883 | 2.04E-02 |
| AE | Thorax, SimCoEx | Blue | asparaginyl-tRNAasn biosynthesis via transamidation | 2 | 6 | 0.2883 | 2.04E-02 |
| AE | Thorax, SimCoEx | Blue | arginine degradation VI (arginase 2 pathway) | 2 | 6 | 0.2883 | 2.04E-02 |
| AE | Thorax, SimCoEx | Blue | isoleucine degradation I | 2 | 6 | 0.2883 | 2.04E-02 |
| C18 | Thorax, SimCoEx | Brown | valine degradation I | 2 | 6 | 0.06129 | 2.19E-02 |
| C18 | Thorax, SimCoEx | Brown | isoleucine degradation I | 2 | 6 | 0.06129 | 2.19E-02 |
| C18 | Thorax, SimCoEx | Brown | 4-hydroxyproline degradation I | 2 | 6 | 0.06129 | 2.19E-02 |
| -- | DR, DiffCoEx | Black | uridine-5'-phosphate biosynthesis | 2 | 9 | 0.0749 | 2.23E-02 |
| -- | DR, DiffCoEx | Green | RNA charging pathway | 2 | 20 | 0.11103 | 2.36E-02 |
| -- | AL, DiffCoEx | Yellow | degradation of purine ribonucleosides | 2 | 8 | 0.03403 | 2.49E-02 |
| -- | DR, DiffCoEx | Black | arginine biosynthesis IV | 2 | 11 | 0.10725 | 2.62E-02 |
| -- | DR, DiffCoEx | Black | formylTHF biosynthesis I | 2 | 12 | 0.12462 | 2.82E-02 |
| C18 | Thorax, DiffCoEx | Turquoise | tRNA charging pathway | 2 | 19 | 0.25244 | 3.02E-02 |
| C18 | Thorax, SimCoEx | Brown | pyrimidine ribonucleotides interconversion | 2 | 8 | 0.10453 | 3.17E-02 |
| C18 | Thorax, SimCoEx | Brown | arginine degradation I (arginase pathway) | 2 | 8 | 0.10453 | 3.17E-02 |
| AE | Thorax, DiffCoEx | Turquoise | glutamine biosynthesis I | 2 | 4 | 0.25175 | 3.35E-02 |
| AE | Thorax, DiffCoEx | Turquoise | PRPP biosynthesis I | 2 | 4 | 0.25175 | 3.35E-02 |
| AE | Thorax, DiffCoEx | Turquoise | 2-methylbutyrate biosynthesis | 2 | 4 | 0.25175 | 3.35E-02 |
| AE | Thorax, DiffCoEx | Turquoise | fatty acid β-oxidation II (core pathway) | 2 | 4 | 0.25175 | 3.35E-02 |
| AE | Thorax, DiffCoEx | Turquoise | fatty acid β-oxidation I | 2 | 4 | 0.25175 | 3.35E-02 |
| AE | Thorax, DiffCoEx | Turquoise | aspartate degradation II | 2 | 4 | 0.25175 | 3.35E-02 |
| AE | Thorax, DiffCoEx | Turquoise | glycine cleavage complex | 2 | 4 | 0.25175 | 3.35E-02 |
| AE | Thorax, DiffCoEx | Turquoise | arginine degradation X (arginine monooxygenase pathway) | 2 | 4 | 0.25175 | 3.35E-02 |
| AE | Thorax, DiffCoEx | Turquoise | aerobic respiration -- electron donor II | 2 | 4 | 0.25175 | 3.35E-02 |
| AE | Thorax, SimCoEx | Blue | tryptophan degradation to 2-amino-3-carboxymuconate semialdehyde | 2 | 8 | 0.42827 | 3.56E-02 |
| AE | Thorax, SimCoEx | Blue | leucine degradation I | 2 | 8 | 0.42827 | 3.56E-02 |
| C18 | Thorax, SimCoEx | Brown | leucine degradation I | 2 | 9 | 0.1285 | 3.76E-02 |
| C18 | Thorax, DiffCoEx | Cyan | D-mannose degradation | 2 | 2 | 0.00613 | 3.88E-02 |
| AE | Thorax, DiffCoEx | Yellow | salvage pathways of adenine, hypoxanthine, and their nucleosides | 2 | 16 | 0.32078 | 4.35E-02 |
| C18 | Thorax, SimCoEx | Brown | proline biosynthesis II | 2 | 10 | 0.15362 | 4.41E-02 |
| C18 | Thorax, SimCoEx | Brown | proline biosynthesis III | 2 | 10 | 0.15362 | 4.41E-02 |
| AE | Thorax, SimCoEx | Blue | uridine-5'-phosphate biosynthesis | 2 | 9 | 0.49286 | 4.48E-02 |
| AE | Thorax, SimCoEx | Blue | de novo biosynthesis of pyrimidine deoxyribonucleotides | 2 | 9 | 0.49286 | 4.48E-02 |
| C18 | Thorax, DiffCoEx | Cyan | myo-inositol biosynthesis | 2 | 3 | 0.01747 | 4.58E-02 |
| C18 | Thorax, DiffCoEx | Cyan | glycogen degradation II | 2 | 3 | 0.01747 | 4.58E-02 |
| C18 | Thorax, DiffCoEx | Cyan | UDP-galactose biosynthesis (salvage pathway from galactose using UDP-glucose) | 2 | 4 | 0.03317 | 5.37E-02 |
| AE | Thorax, SimCoEx | Cyan | fatty acid β-oxidation II (core pathway) | 2 | 4 | 0.00124 | 5.43E-02 |
| AE | Thorax, SimCoEx | Cyan | fatty acid β-oxidation I | 2 | 4 | 0.00124 | 5.43E-02 |
| AE | Thorax, SimCoEx | Cyan | oxidative ethanol degradation III | 2 | 5 | 0.00205 | 5.61E-02 |
| AE | Thorax, SimCoEx | Cyan | oxidative ethanol degradation I | 2 | 5 | 0.00205 | 5.61E-02 |
| AE | Thorax, SimCoEx | Cyan | 4-hydroxyproline degradation I | 2 | 5 | 0.00205 | 5.61E-02 |
| C18 | Thorax, SimCoEx | Pink | coenzyme A biosynthesis | 2 | 7 | 0.01777 | 6.04E-02 |
| -- | AL, DiffCoEx | Cyan | methyl parathion degradation | 2 | 2 | 0.00017 | 6.41E-02 |
| -- | AL, DiffCoEx | LightCyan | uridine-5'-phosphate biosynthesis | 2 | 9 | 0.01403 | 6.60E-02 |
| C18 | Thorax, SimCoEx | Pink | salvage pathways of pyrimidine ribonucleotides | 2 | 9 | 0.02933 | 6.85E-02 |
| C18 | Thorax, SimCoEx | Pink | de novo biosynthesis of pyrimidine deoxyribonucleotides | 2 | 9 | 0.02933 | 6.85E-02 |
| C18 | Thorax, DiffCoEx | Pink | glycogen degradation I | 2 | 10 | 0.02594 | 7.24E-02 |
| AE | Thorax, DiffCoEx | Yellow | tRNA charging pathway | 2 | 20 | 0.42774 | 7.30E-02 |
| AE | Thorax, SimCoEx | Green | β-alanine betaine biosynthesis | 2 | 6 | 0.01003 | 8.24E-02 |
| -- | DR, DiffCoEx | Magenta | 2-methylbutyrate biosynthesis | 2 | 4 | 0.00042 | 8.77E-02 |
| -- | DR, DiffCoEx | Magenta | oxidative ethanol degradation III | 2 | 5 | 0.00069 | 8.91E-02 |
| -- | DR, DiffCoEx | Magenta | oxidative ethanol degradation I | 2 | 5 | 0.00069 | 8.91E-02 |
| AE | Thorax, DiffCoEx | Magenta | myo-inositol biosynthesis | 2 | 2 | 0.00346 | 0.09642 |
| AE | Thorax, DiffCoEx | Magenta | D-mannose degradation | 2 | 2 | 0.00346 | 0.09642 |
| AE | Thorax, DiffCoEx | Magenta | glycogen degradation II | 2 | 2 | 0.00346 | 0.09642 |
| AE | Thorax, DiffCoEx | Black | glutathione redox reactions II | 2 | 3 | 0.001656766 | 0.096676428 |
| AE | Thorax, DiffCoEx | Black | glutathione redox reactions I | 2 | 3 | 0.001656766 | 9.67E-02 |
| -- | AL, DiffCoEx | Black | choline biosynthesis III | 2 | 4 | 0.03224 | 9.92E-02 |
| -- | DR, DiffCoEx | DarkRed | salvage pathways of adenine, hypoxanthine, and their nucleosides | 2 | 16 | 0.06147 | 9.94E-02 |
| -- | AL, DiffCoEx | Black | phosphatidylethanolamine biosynthesis II | 2 | 5 | 0.05108 | 1.18E-01 |
| -- | AL, DiffCoEx | Black | proline degradation I | 2 | 5 | 0.05108 | 1.18E-01 |
| -- | AL, DiffCoEx | Black | 4-hydroxyproline degradation I | 2 | 5 | 0.05108 | 1.18E-01 |
| AE | Thorax, DiffCoEx | Magenta | UDP-galactose biosynthesis (salvage pathway from galactose using UDP-glucose) | 2 | 4 | 0.01922 | 0.12147 |
| AE | Thorax, DiffCoEx | Magenta | GDP-mannose biosynthesis II | 2 | 5 | 0.03084 | 0.13511 |
| -- | AL, DiffCoEx | Black | 4-hydroxybenzoate biosynthesis I (eukaryotes) | 2 | 6 | 0.07285 | 1.38E-01 |

Table S5: Normalized Metabolite Levels from Metabolon Data Set

| **BIOCHEMICAL** | **MASS** | **10d DR** | **10d AL** | **p-value** |
| --- | --- | --- | --- | --- |
| 1,3-dihydroxyacetone | 306.1 | 1.43016 | 0.76812 | 0.007 |
| 1-eicosenoylglycerophosphoethanolamine (20:1n9)* | 506.5 | 1.21678 | 1.10896 | 0.6812 |
| 1-linolenoylglycerophosphocholine (18:3n3)* | 518.4 | 1.2505 | 0.54172 | 0.0005 |
| 1-linolenoylglycerophosphoethanolamine (18:3n3)* | 474.4 | 1.2946 | 0.64682 | 7.7636E-06 |
| 1-linolenoylglycerophosphoinositol* | 593.2 | 1.16214 | 0.49898 | 0.000063449 |
| 1-linolenoylglycerophosphoserine* | 518.3 | 1.68324 | 0.44148 | 3.1244E-06 |
| 1-linoleoylglycerophosphocholine (18:2n6) | 520.4 | 1.38906 | 0.57734 | 0.0009 |
| 1-linoleoylglycerophosphoethanolamine* | 476.3 | 1.6713 | 0.80648 | 0.000045522 |
| 1-linoleoylglycerophosphoinositol* | 595.3 | 1.18524 | 0.64718 | 7.2563E-06 |
| 1-linoleoylglycerophosphoserine* | 520.3 | 2.21374 | 0.67556 | 2.1306E-06 |
| 1-myristoleoylglycerophosphocholine (14:1) * | 466.3 | 0.92586 | 1.196 | 0.2327 |
| 1-myristoleoylglycerophosphoinositol* | 541.4 | 0.66426 | 1.42542 | 0.000012118 |
| 1-myristoylglycerophosphocholine (14:0) | 468.3 | 0.84382 | 1.1827 | 0.0609 |
| 1-myristoylglycerophosphoethanolamine | 424.3 | 0.65576 | 1.51658 | 1.5399E-06 |
| 1-octadecanol | 327.3 | 1.39722 | 0.72204 | 0.0002 |
| 1-oleoylglycerophosphate | 637.5 | 1.16602 | 1.0566 | 0.6103 |
| 1-oleoylglycerophosphocholine (18:1) | 522.4 | 1.08356 | 0.8026 | 0.0481 |
| 1-oleoylglycerophosphoethanolamine | 478.3 | 1.10684 | 0.84556 | 0.0165 |
| 1-oleoylglycerophosphoglycerol* | 509.5 | 0.86514 | 0.8972 | 0.7227 |
| 1-oleoylglycerophosphoinositol* | 597.4 | 0.78158 | 1.07024 | 0.0097 |
| 1-oleoylglycerophosphoserine | 522.3 | 1.182 | 0.57776 | 0.000019583 |
| 1-oleoylplasmenylethanolamine* | 462.5 | 0.95304 | 0.57574 | 0.4927 |
| 1-palmitoleoylglycerophosphocholine (16:1)* | 494.3 | 0.95094 | 0.95902 | 0.958 |
| 1-palmitoleoylglycerophosphoethanolamine* | 450.4 | 0.94384 | 1.04622 | 0.1016 |
| 1-palmitoleoylglycerophosphoinositol* | 569.3 | 0.95206 | 1.08304 | 0.0899 |
| 1-palmitoylglycerol (1-monopalmitin) | 371.3 | 1.19858 | 0.88208 | 0.0339 |
| 1-palmitoylglycerophosphocholine (16:0) | 496.4 | 1.187 | 0.8605 | 0.0208 |
| 1-palmitoylglycerophosphoethanolamine | 452.3 | 1.08198 | 1.2551 | 0.2565 |
| 1-palmitoylglycerophosphoglycerol* | 483.4 | 1.11968 | 0.96402 | 0.0839 |
| 1-palmitoylglycerophosphoinositol* | 571.3 | 1.01644 | 1.06596 | 0.7165 |
| 1-palmitoylplasmenylethanolamine* | 436.4 | 1.35504 | 1.12172 | 0.3967 |
| 1-stearoylglycerol (1-monostearin) | 399.4 | 1.06974 | 0.75474 | 0.3033 |
| 1-stearoylglycerophosphocholine (18:0) | 524.4 | 1.38132 | 1.05302 | 0.2344 |
| 1-stearoylglycerophosphoethanolamine | 480.4 | 1.0882 | 1.25504 | 0.5615 |
| 1-stearoylglycerophosphoglycerol | 511.4 | 0.8022 | 0.99314 | 0.1408 |
| 1-stearoylglycerophosphoinositol | 599.4 | 0.90138 | 1.10974 | 0.0356 |
| 1-stearoylglycerophosphoserine* | 524.4 | 1.15242 | 0.67586 | 0.0006 |
| 1-stearoylplasmenylethanolamine* | 464.4 | 1.04356 | 1.09588 | 0.876 |
| 10-heptadecenoate (17:1n7) | 267.3 | 1.2271 | 0.64476 | 0.000007623 |
| 10-nonadecenoate (19:1n9) | 295.4 | 0.9786 | 0.98958 | 0.8659 |
| 13-HODE + 9-HODE | 295.2 | 1.88078 | 0.66494 | 0.000014273 |
| 15-methylpalmitate (isobar with 2-methylpalmitate) | 269.4 | 2.06962 | 0.57576 | 4.1566E-09 |
| 17-methylstearate | 297.4 | 1.96006 | 0.42568 | 1.4133E-07 |
| 2'-deoxyguanosine | 266.2 | 0.64764 | 0.9859 | 0.000018882 |
| 2'-deoxyinosine | 251.1 | 0.72032 | 1.18792 | 6.4769E-08 |
| 2-aminobutyrate | 130 | 0.89998 | 1.93274 | 0.000093976 |
| 2-aminooctanoate | 160.1 | 1.5588 | 0.5455 | 0.0005 |
| 2-hydroxyglutarate | 247 | 0.74058 | 1.52506 | 0.0002 |
| 2-linolenoylglycerophosphocholine(18:3n3)* | 518.3 | 1.50898 | 0.46464 | 0.0006 |
| 2-linolenoylglycerophosphoethanolamine (18:3n3)* | 476.2 | 1.41908 | 0.47074 | 0.3708 |
| 2-linolenoylglycerophosphoinositol* | 593.2 | 1.30094 | 0.5834 | 0.000027136 |
| 2-linoleoylglycerol (2-monolinolein) | 408.3 | 1.06822 | 0.74718 | 2.9049E-06 |
| 2-linoleoylglycerophosphocholine* | 520.4 | 1.93148 | 0.60056 | 0.7057 |
| 2-linoleoylglycerophosphoethanolamine* | 476.4 | 1.91676 | 0.7193 | 8.3901E-07 |
| 2-linoleoylglycerophosphoinositol* | 595.3 | 0.92754 | 0.8974 | 0.8791 |
| 2-methylcitrate | 287.1 | 2.29154 | 0.77574 | 0.2493 |
| 2-myristoleoylglycerophosphocholine* | 466.3 | 1.0966 | 1.097 | 0.0003 |
| 2-myristoylglycerophosphocholine* | 468.3 | 0.84818 | 1.1261 |  |
| 2-myristoylglycerophosphoethanolamine* | 424.2 | 0.69434 | 1.3953 | 0.0191 |
| 2-oleoylglycerol (2-monoolein) | 410.1 | 0.6465 | 0.6465 | 0.0222 |
| 2-oleoylglycerophosphocholine* | 596 | 1.09958 | 0.77264 | 0.0079 |
| 2-oleoylglycerophosphoethanolamine* | 478.3 | 1.05212 | 0.81128 | 0.0006 |
| 2-oleoylglycerophosphoinositol* | 597.5 | 0.80832 | 1.11278 | 0.0831 |
| 2-oleoylglycerophosphoserine* | 522.3 | 1.11994 | 0.64856 | 0.185 |
| 2-palmitoleoylglycerophosphocholine* | 494.3 | 1.1694 | 0.92868 | 0.0001 |
| 2-palmitoleoylglycerophosphoethanolamine* | 450.3 | 0.9833 | 1.0799 | 0.0026 |
| 2-palmitoleoylglycerophosphoinositol* | 569.3 | 0.85736 | 1.3133 | 0.6229 |
| 2-palmitoylglycerophosphocholine* | 496.3 | 1.31184 | 0.71448 | 0.0979 |
| 2-palmitoylglycerophosphoethanolamine* | 452.3 | 0.99402 | 1.06962 | 0.0681 |
| 2-palmitoylglycerophosphoglycerol* | 483.4 | 1.30102 | 0.86874 | 0.0219 |
| 2-phosphoglycerate | 459.1 | 2.64046 | 1.51828 | 0.0061 |
| 2-pyrrolidinone | 142 | 1.6521 | 1.15426 | 0.3765 |
| 2-stearoylglycerophosphocholine* | 524.4 | 1.3028 | 0.67092 | 0.5732 |
| 2-stearoylglycerophosphoglycerol* | 511.4 | 0.79826 | 0.94848 | 0.0008 |
| 2-stearoylglycerophosphoinositol* | 599.4 | 0.93346 | 1.03248 | 0.0004 |
| 3'-dephosphocoenzyme A | 686.2 | 0.9097 | 1.2693 | 0.0014 |
| 3,4-dihydroxyphenylacetate | 384.1 | 0.39458 | 1.49776 | 0.0025 |
| 3-aminoisobutyrate | 102 | 1.23856 | 1.5841 | 0.0028 |
| 3-dehydrocarnitine* | 160.2 | 1.1679 | 1.57978 | 0.0419 |
| 3-hydroxy-2-ethylpropionate | 247.1 | 1.26008 | 0.17446 | 0.0006 |
| 3-hydroxy-3-methylglutarate | 247 | 0.32002 | 1.50654 | 0.000029178 |
| 3-hydroxydecanoate | 187.2 | 1.12748 | 1.06568 | 0.6477 |
| 3-hydroxyoctanoate | 159.2 | 0.99662 | 1.04844 | 0.5551 |
| 3-hydroxypropanoate | 177.1 | 2.48236 | 0.64136 | 8.9062E-07 |
| 3-hydroxysebacate | 217.2 | 1.70072 | 1.01772 | 0.8926 |
| 3-methyl-2-oxobutyrate | 115.1 | 1.70322 | 1.56018 | 0.6206 |
| 3-methyl-2-oxovalerate | 129.2 | 2.23226 | 1.58226 | 0.1299 |
| 3-phosphoglycerate | 299 | 2.2634 | 1.13576 | 0.0006 |
| 3-ureidopropionate | 133.1 | 1.3179 | 0.83584 | 0.0015 |
| 4-hydroxy-2-oxoglutaric acid | 333.1 | 5.77384 | 0.16098 | 0.0002 |
| 4-hydroxybutyrate (GHB) | 233.1 | 2.09768 | 0.85192 | 0.0048 |
| 4-methyl-2-oxopentanoate | 129.2 | 3.2285 | 1.63542 | 0.0308 |
| 5,6-dihydrouracil | 243 | 0.74674 | 1.17508 | 0.0078 |
| 5-aminovalerate | 118.1 | 0.57036 | 1.3183 | 0.0034 |
| 5-dodecenoate (12:1n7) | 197.2 | 1.65896 | 1.06934 | 0.000014157 |
| 5-methylthioadenosine (MTA) | 298.1 | 0.74486 | 1.09382 | 0.0022 |
| 5-oxoproline | 128.2 | 0.90814 | 1.21644 | 0.0014 |
| 7-beta-hydroxycholesterol | 456.4 | 3.15892 | 0.8105 | 0.0013 |
| 9,10-DiHOME | 313.3 | 1.1896 | 0.93302 | 0.0398 |
| 9-methyluric acid | 383.1 | 0.0289 | 0.70348 | 0.0162 |
| acetyl CoA | 808.1 | 0.5667 | 1.10218 | 0.0045 |
| acetylcarnitine | 204.2 | 1.51114 | 0.86566 | 0.0093 |
| acetylcholine | 146.1 | 0.69072 | 1.17174 | 0.119 |
| adenine | 136.1 | 1.09596 | 0.54144 | 0.0012 |
| adenosine | 268.1 | 1.2167 | 0.45004 | 0.0008 |
| adenosine 3'-monophosphate (3'-AMP) | 346.1 | 1.08396 | 1.17544 | 0.2264 |
| adenosine 5'-diphosphate (ADP) | 426.1 | 1.58168 | 0.90432 | 0.0001 |
| adenosine 5'-monophosphate (AMP) | 348.1 | 0.98624 | 0.38818 | 0.0252 |
| adenosine-2',3'-cyclic monophosphate | 328.2 | 0.9575 | 0.98442 | 0.7226 |
| adenylosuccinate | 462.1 | 0.71238 | 0.502 | 0.3351 |
| alanine | 115.9 | 1.29236 | 1.25634 | 0.6795 |
| alanylalanine | 271.1 | 1.01446 | 1.04716 | 0.769 |
| alanylleucine | 203 | 2.26602 | 1.1331 | 0.0044 |
| alanylphenylalanine | 237.2 | 1.99226 | 1.11828 | 0.0001 |
| alanylproline | 187.1 | 2.19934 | 1.16622 | 0.000080336 |
| alanyltyrosine | 253 | 1.12642 | 1.18824 | 0.5538 |
| alanylvaline | 189 | 1.8723 | 1.19658 | 0.1207 |
| allantoin | 518.3 | 0.51884 | 1.64786 | 0.0351 |
| alpha-hydroxyisocaproate | 131.2 | 0.88542 | 1.3493 | 0.1066 |
| alpha-hydroxyisovalerate | 145.1 | 0.4099 | 1.16756 | 0.0016 |
| alpha-ketoglutarate | 347.1 | 19.51274 | 1.11384 | 0.0004 |
| arabitol | 307.1 | 0.52226 | 3.59874 | 2.6235E-06 |
| arabonate | 292.1 | 0.80816 | 1.08784 | 0.022 |
| arachidate (20:0) | 311.3 | 1.01802 | 0.95846 | 0.2623 |
| arachidyl alcohol | 355.3 | 1.18866 | 0.27894 | 0.000023078 |
| arginine | 173.2 | 1.47774 | 0.97 | 0.0011 |
| ascorbate (Vitamin C) | 332.1 | 0.24292 | 1.15446 | 0.000051792 |
| asparagine | 133.1 | 1.37524 | 1.21946 | 0.1962 |
| asparagylisoleucine | 246.2 | 2.16198 | 1.10234 | 0.0084 |
| asparagylleucine | 246.2 | 2.42582 | 1.25524 | 0.0005 |
| aspartate | 232 | 0.29906 | 1.3627 | 0.0023 |
| azelate (nonanedioate) | 187.2 | 1.18716 | 0.76672 | 0.0503 |
| beta-alanine | 174 | 1.8693 | 0.92984 | 0.026 |
| beta-hydroxypyruvate | 221 | 1.30876 | 0.75166 | 0.0921 |
| beta-sitosterol | 357.4 | 1.49896 | 0.57512 | 6.9981E-09 |
| betaine | 118.2 | 0.88852 | 1.74216 | 0.0001 |
| biopterin | 238.1 | 1.92888 | 0.88224 | 0.0006 |
| biotin | 245.2 | 0.5502 | 1.14776 | 0.0008 |
| campesterol | 343.4 | 1.78112 | 0.7622 | 0.0002 |
| caprate (10:0) | 171.2 | 1.3826 | 1.04852 | 0.0117 |
| caproate (6:0) | 115.2 | 1.37074 | 0.87988 | 0.1466 |
| caprylate (8:0) | 143.2 | 1.2403 | 1.20976 | 0.748 |
| carnitine | 162.2 | 2.12644 | 0.73436 | 8.9106E-07 |
| choline phosphate | 184.1 | 1.29108 | 0.5319 | 0.000033376 |
| cis-vaccenate (18:1n7) | 339.3 | 1.04496 | 1.06338 | 0.7752 |
| citrate | 273.1 | 2.15028 | 0.77724 | 0.000016335 |
| citrulline | 176.1 | 1.04786 | 1.61748 | 0.0004 |
| coenzyme A | 766.1 | 0.77992 | 1.45594 | 0.0022 |
| cyclic adenosine diphosphate-ribose | 542 | 1.5559 | 0.97014 | 0.0004 |
| cyclo(gly-pro) | 155.1 | 0.68648 | 1.2636 | 0.0094 |
| cyclo(leu-pro) | 211.1 | 0.58136 | 1.09932 | 0.0011 |
| cysteine | 218 | 0.43366 | 1.28716 | 0.0232 |
| cytidine | 244 | 1.04806 | 2.67318 | 8.5658E-07 |
| cytidine 5'-monophosphate (5'-CMP) | 324 | 0.22168 | 0.8408 | 4.1983E-06 |
| cytosine-2',3'-cyclic monophosphate | 304.1 | 0.77368 | 2.04262 | 0.000047576 |
| dihomo-linoleate (20:2n6) | 307.3 | 0.91456 | 1.14486 | 0.1205 |
| dihydrobiopterin | 240.1 | 0.77264 | 1.16726 | 0.0598 |
| dimethylarginine (SDMA + ADMA) | 203.2 | 1.37516 | 1.188 | 0.1658 |
| docosadienoate (22:2n6) | 335.4 | 1.00076 | 1.3199 | 0.017 |
| dodecanedioate | 229.2 | 1.03166 | 1.41984 | 0.0597 |
| eicosenoate (20:1n9 or 11) | 309.4 | 1.19712 | 0.9004 | 0.0252 |
| ergosterol | 363.3 | 1.28174 | 0.67948 | 0.0001 |
| erlose | 361.2 | 2.5189 | 0.13954 | 0.000025256 |
| erucate (22:1n9) | 337.4 | 1.3153 | 0.97964 | 0.0062 |
| erythritol | 217 | 1.14354 | 1.001 | 0.3701 |
| erythronate* | 292.1 | 1.0335 | 0.98462 | 0.6801 |
| ethanolamine | 102 | 0.76494 | 1.62658 | 0.0148 |
| flavin adenine dinucleotide (FAD) | 784.1 | 0.8357 | 1.12128 | 0.0018 |
| flavin mononucleotide (FMN) | 455.1 | 1.04518 | 1.0941 | 0.5375 |
| fructose | 217 | 2.15598 | 0.47858 | 0.000018561 |
| fructose-6-phosphate | 315.1 | 2.10134 | 0.61726 | 4.5518E-06 |
| fumarate | 245 | 0.99192 | 0.93346 | 0.3988 |
| gamma-aminobutyrate (GABA) | 304.1 | 1.96986 | 0.84704 | 0.0029 |
| gamma-glutamylisoleucine* | 261.2 | 1.13968 | 1.6571 | 0.0667 |
| gamma-glutamylleucine | 261.2 | 1.36386 | 1.55706 | 0.0477 |
| gamma-glutamylphenylalanine | 295.1 | 1.08538 | 1.22338 | 0.4148 |
| gamma-glutamylvaline | 247.2 | 0.85012 | 1.86308 | 0.0298 |
| gluconate | 333 | 0.40718 | 1.94924 | 0.0001 |
| glucose | 217.1 | 1.189 | 1.01578 | 0.0335 |
| glucose 1-phosphate | 299.1 | 1.4521 | 0.43208 | 0.0021 |
| glucose-6-phosphate (G6P) | 387.2 | 2.2938 | 0.513 | 5.7224E-07 |
| glutamate | 148.1 | 1.4062 | 1.11134 | 0.0167 |
| glutamine | 147.2 | 1.47686 | 1.05162 | 0.0072 |
| glutamine-leucine | 260.2 | 2.26388 | 1.15198 | 0.002 |
| glutarate (pentanedioate) | 261 | 0.92048 | 3.04706 | 0.0007 |
| glycerate | 189 | 1.49838 | 0.94066 | 0.0057 |
| glycerol | 205 | 1.0804 | 1.03956 | 0.5057 |
| glycerol 2-phosphate | 243 | 1.03264 | 0.95344 | 0.2005 |
| glycerol 3-phosphate (G3P) | 357.1 | 0.46062 | 1.26298 | 0.000058565 |
| glycerophosphoethanolamine | 357.1 | 0.08432 | 5.81342 | 0.0002 |
| glycerophosphorylcholine (GPC) | 258.1 | 1.42912 | 0.74452 | 0.000010874 |
| glycine | 101.9 | 1.20978 | 1.16078 | 0.4536 |
| glycylisoleucine | 189.1 | 1.90012 | 1.12116 | 0.0026 |
| glycylleucine | 189.1 | 2.19372 | 1.1372 | 0.000045678 |
| glycylphenylalanine | 221.2 | 2.13266 | 1.11216 | 0.0000089 |
| glycylproline | 173.1 | 1.4378 | 1.07014 | 0.0001 |
| glycyltryptophan | 262.4 | 1.5272 | 1.0492 | 0.0053 |
| glycylvaline | 175.1 | 1.55964 | 1.07082 | 0.0007 |
| guanosine | 284 | 0.81806 | 1.0383 | 0.0031 |
| guanosine 5'- monophosphate (5'-GMP) | 364 | 0.73524 | 0.54402 | 0.2603 |
| guanosine 5'-diphospho-fucose | 588.1 | 1.31496 | 1.06962 | 0.1439 |
| guanosine-2',3'-cyclic monophosphate | 343.9 | 0.99876 | 0.99876 | 0.9873 |
| gulono-1,4-lactone | 333.1 | 0.67628 | 1.2682 | 0.000059575 |
| heptanoate (7:0) | 129.2 | 1.224 | 1.0256 | 0.2746 |
| hexadecanedioate | 285.2 | 1.09342 | 0.95058 | 0.4496 |
| histamine | 112.2 | 2.21728 | 0.97884 | 0.0006 |
| histidine | 154.1 | 1.12036 | 1.02748 | 0.0633 |
| homocitrulline | 190.1 | 0.8932 | 1.36918 | 0.003 |
| homocysteine | 234 | 0.52848 | 1.105 | 0.0003 |
| homoserine | 218.1 | 1.3063 | 1.17978 | 0.3609 |
| hypoxanthine | 135.1 | 0.8851 | 1.67128 | 0.0169 |
| inosine | 267.2 | 1.27662 | 0.99622 | 0.000012794 |
| inosine 5'-monophosphate (IMP) | 349 | 0.9417 | 0.26488 | 0.0078 |
| inositol 1-phosphate (I1P) | 318.1 | 1.05694 | 1.06522 | 0.865 |
| Isobar: fructose 1,6-diphosphate, glucose 1,6-diphosphate, myo-inositol 1,4 or 1,3-diphosphate | 339 | 1.68312 | 0.73472 | 0.0029 |
| Isobar: pentulose 5-phosphates | 357.2 | 1.87002 | 0.69576 | 0.0067 |
| isobutyrylcarnitine | 232.2 | 2.27042 | 0.96866 | 0.0047 |
| isoleucine | 132.1 | 1.20438 | 1.1826 | 0.7541 |
| isoleucylaspartate | 247.2 | 1.67808 | 1.15916 | 0.000091754 |
| isoleucylglycine | 189.2 | 1.97968 | 1.08656 | 0.000085783 |
| isomaltose | 204.1 | 0.72442 | 1.1508 | 0.0014 |
| kynurenate | 188.1 | 1.1258 | 1.61024 | 0.1161 |
| kynurenine | 209.1 | 0.63528 | 1.82468 | 3.8253E-07 |
| lactate | 116.9 | 0.81084 | 1.30856 | 0.0013 |
| laurate (12:0) | 199.3 | 1.378 | 1.02996 | 0.0014 |
| leucine | 132.2 | 1.1743 | 1.13126 | 0.5158 |
| leucylaspartate | 247.2 | 1.64786 | 1.21382 | 0.0161 |
| leucylglycine | 189.2 | 1.68824 | 1.11778 | 0.0881 |
| linoleate (18:2n6) | 279.3 | 1.5778 | 0.96518 | 2.5432E-06 |
| linolenate [alpha or gamma; (18:3n3 or 6)] | 277.3 | 1.27928 | 0.5372 | 1.8046E-06 |
| lysine | 147.2 | 1.25054 | 1.07118 | 0.0558 |
| lysylisoleucine | 258.3 | 1.99288 | 0.99984 | 0.000067954 |
| lysylleucine | 260.2 | 2.77574 | 1.10204 | 0.000077682 |
| malate | 233 | 1.48056 | 0.78466 | 0.0013 |
| maltohexaose | 989.1 | 1.18978 | 0.8052 | 0.0013 |
| maltopentaose | 827.1 | 1.35048 | 0.2694 | 5.7804E-06 |
| maltose | 204.1 | 2.60266 | 0.43184 | 0.000055085 |
| maltotetraose | 665.1 | 1.3363 | 0.6164 | 0.000084757 |
| maltotriose | 204 | 1.81678 | 0.48958 | 0.000029265 |
| mannitol | 319.1 | 0.65518 | 1.859 | 0.0005 |
| mannose | 204 | 2.09198 | 0.8035 | 1.3915E-06 |
| mannose-6-phosphate | 387.2 | 2.4672 | 0.59238 | 1.7391E-06 |
| margarate (17:0) | 269.3 | 1.13484 | 0.87022 | 0.1008 |
| methionine | 150.1 | 1.29356 | 1.09154 | 0.0075 |
| methionine sulfoxide | 166.1 | 0.68388 | 1.01346 | 0.0056 |
| methyl-alpha-glucopyranoside | 204 | 1.45414 | 0.7773 | 0.0104 |
| methylphosphate | 241 | 1.23346 | 0.7978 | 0.0035 |
| myo-inositol | 217 | 0.96668 | 1.04424 | 0.2301 |
| myristate (14:0) | 227.3 | 1.19288 | 1.07522 | 0.0013 |
| myristoleate (14:1n5) | 225.3 | 1.26684 | 1.20264 | 0.2512 |
| N-acetylalanine | 130.1 | 0.88578 | 1.3408 | 0.0377 |
| N-acetylarginine | 217.2 | 0.4975 | 1.36506 | 0.0001 |
| N-acetylaspartate (NAA) | 157.9 | 4.5784 | 0.7106 | 0.000048208 |
| N-acetylleucine | 174.1 | 0.76772 | 1.08472 | 0.0799 |
| N-acetylmethionine | 190.1 | 1.20048 | 1.22546 | 0.8146 |
| N-acetylphenylalanine | 206.2 | 0.91366 | 1.04348 | 0.2086 |
| N-acetylserine | 218 | 0.79192 | 1.09658 | 0.2372 |
| N-methylalanine | 129.9 | 1.55962 | 0.85034 | 0.0001 |
| N1-methyladenosine | 282.1 | 0.9121 | 1.1661 | 0.0209 |
| N1-methylguanosine | 298.1 | 0.81106 | 1.62384 | 0.0104 |
| N2-acetyllysine | 189.1 | 0.80628 | 1.54076 | 0.000067287 |
| N6-acetyllysine | 189.1 | 1.15182 | 1.22412 | 0.3285 |
| N6-carbamoylthreonyladenosine | 413 | 1.04376 | 0.98436 | 0.7031 |
| nicotinamide | 123.1 | 1.24342 | 1.2606 | 0.904 |
| nicotinamide adenine dinucleotide (NAD+) | 664 | 1.1952 | 0.8588 | 0.0007 |
| nicotinamide adenine dinucleotide phosphate reduced (NADPH) | 744.1 | 0.51594 | 1.53744 | 0.0001 |
| nicotinamide adenine dinucleotide reduced (NADH) | 664.1 | 0.71534 | 1.00478 | 0.0212 |
| nicotinamide ribonucleotide (NMN) | 335.1 | 1.06332 | 0.97028 | 0.4429 |
| nicotinamide riboside* | 255.1 | 1.54044 | 1.3798 | 0.3617 |
| nicotinate | 180 | 1.0873 | 1.1427 | 0.5501 |
| nicotinate ribonucleoside* | 256 | 0.94992 | 1.19568 | 0.1157 |
| nonadecanoate (19:0) | 297.3 | 0.91314 | 1.1792 | 0.0458 |
| oleate (18:1n9) | 339.2 | 1.04336 | 0.88744 | 0.0155 |
| oleoylcarnitine | 426.4 | 2.28732 | 0.7716 | 0.0001 |
| ornithine | 142 | 1.5434 | 1.70308 | 0.9121 |
| palmitate (16:0) | 255.3 | 1.2693 | 1.03932 | 0.0006 |
| palmitoleate (16:1n7) | 253.3 | 1.06688 | 1.02244 | 0.3573 |
| palmitoylcarnitine | 400.4 | 2.00934 | 0.8276 | 0.0059 |
| panose | 204.1 | 1.1853 | 0.7017 | 0.0117 |
| pantethine | 553.3 | 1.14832 | 1.4053 | 0.0227 |
| pantothenate | 220.1 | 0.74744 | 1.36326 | 0.00007192 |
| pelargonate (9:0) | 157.2 | 1.18484 | 0.77814 | 0.0013 |
| pentadecanoate (15:0) | 299.2 | 1.41414 | 0.87958 | 0.000027749 |
| phenylalanine | 166.1 | 1.25782 | 1.17166 | 0.2971 |
| phenyllactate (PLA) | 165.1 | 0.8456 | 0.83372 | 0.9095 |
| phosphate | 298.9 | 1.09434 | 1.02418 | 0.2832 |
| phosphoenolpyruvate (PEP) | 369 | 2.45724 | 1.57788 | 0.0202 |
| phosphoethanolamine | 299.1 | 0.85268 | 1.00054 | 0.065 |
| phosphopantetheine | 359.1 | 0.91466 | 1.2141 | 0.0101 |
| pipecolate | 130.1 | 1.41738 | 1.34038 | 0.259 |
| pro-hydroxy-pro | 229.2 | 1.22288 | 1.17212 | 0.7951 |
| proline | 116.1 | 1.28128 | 1.08078 | 0.0241 |
| prolylalanine | 187.1 | 1.96258 | 1.24138 | 0.0004 |
| prolylglutamine | 244.2 | 1.19704 | 1.5086 | 0.045 |
| prolylleucine | 227.2 | 2.37004 | 0.94918 | 0.000020424 |
| prolylproline | 213.1 | 1.8155 | 1.0713 | 0.000035068 |
| prolyltryptophan | 302.2 | 2.31524 | 1.00696 | 0.0004 |
| propionylcarnitine | 218.2 | 3.3527 | 0.34248 | 0.000015237 |
| propionylglycine | 132.1 | 1.84338 | 0.41876 | 0.0006 |
| pseudouridine | 243.1 | 1.04792 | 1.5896 | 0.0016 |
| putrescine | 174 | 1.28528 | 0.63374 | 0.0205 |
| pyridoxal | 168.1 | 0.81448 | 1.25922 | 0.0077 |
| pyridoxate | 182.1 | 0.76958 | 2.60046 | 9.9618E-08 |
| pyroglutamine* | 129.2 | 1.27898 | 1.04908 | 0.3511 |
| pyroglutamylvaline | 227.2 | 0.73386 | 2.0042 | 0.000099685 |
| pyrophosphate (PPi) | 451 | 1.51792 | 1.42364 | 0.6754 |
| pyruvate | 217 | 12.74794 | 0.96366 | 0.00004454 |
| ribitol | 217 | 0.7397 | 1.73936 | 0.0003 |
| riboflavin (Vitamin B2) | 377.2 | 0.49278 | 1.60048 | 7.0276E-07 |
| ribose | 204 | 1.07586 | 1.16286 | 0.6708 |
| ribulose | 306.1 | 0.61916 | 2.5569 | 0.0006 |
| S-adenosylhomocysteine (SAH) | 193.2 | 0.7321 | 1.04148 | 0.0499 |
| sebacate (decanedioate) | 201.2 | 1.13728 | 1.2619 | 0.4577 |
| sedoheptulose-7-phosphate | 691.35 | 1.59488 | 0.8177 | 0.0002 |
| serine | 204 | 1.21508 | 1.0751 | 0.0484 |
| serylleucine | 219.2 | 1.78222 | 1.16536 | 0.0016 |
| sorbitol | 319.1 | 0.75436 | 1.0933 | 0.0046 |
| spermidine | 146.2 | 1.43316 | 0.96096 | 0.0018 |
| sphinganine | 302.3 | 0.67508 | 1.03696 | 0.0717 |
| stearate (18:0) | 283.4 | 1.158 | 0.8205 | 0.0007 |
| stearidonate (18:4n3) | 275.3 | 1.17468 | 0.54778 | 0.0014 |
| stearoylcarnitine | 428.4 | 1.09062 | 0.87162 | 0.1712 |
| succinate | 247 | 0.67854 | 1.26432 | 0.0018 |
| tagatose | 217.1 | 1.09922 | 1.02366 | 0.1723 |
| taurine | 254.1 | 1.72028 | 0.49802 | 3.9785E-06 |
| tetradecanedioate | 257.2 | 0.9075 | 1.3463 | 0.003 |
| threitol | 217.1 | 0.91278 | 0.80942 | 0.9637 |
| threonine | 120.1 | 0.78216 | 1.24068 | 0.00004757 |
| threonylleucine | 233.2 | 2.20914 | 0.93394 | 0.001 |
| thymine | 255 | 0.84562 | 1.33134 | 0.0621 |
| trehalose | 361.2 | 0.32302 | 1.37586 | 0.0006 |
| tryptophan | 205.1 | 1.2009 | 1.10626 | 0.1489 |
| tyrosine | 182.1 | 1.17104 | 1.91456 | 0.0432 |
| uracil | 241 | 0.85494 | 1.64256 | 0.0003 |
| urate | 167.1 | 0.22834 | 1.98866 | 1.2496E-09 |
| uridine | 243.1 | 1.12568 | 0.99558 | 0.1079 |
| uridine-2',3'-cyclic monophosphate | 304.9 | 0.85188 | 1.1989 | 0.0002 |
| valine | 118.1 | 1.0692 | 1.2625 | 0.0112 |
| xanthine | 153.1 | 1.0842 | 1.45402 | 0.0445 |
| xanthosine | 285 | 1.13914 | 1.26886 | 0.431 |
| xylitol | 307.2 | 1.13522 | 1.01966 | 0.2821 |
| xylonate | 292 | 0.299 | 2.0523 | 9.1476E-06 |
| xylulose | 306.1 | 2.07864 | 0.85964 | 0.000074214 |

**Figure Legends for Supplemental Figures**

Figure S1: Data from C18 column. **A.** Venn diagram of numbers of significantly (p<0.01) changed metabolites in head, abdomen, and thorax by 10 days of DR. . **B.** Venn diagram of numbers of significantly (p<0.01) changed metabolites at 10, 20, and 40 days of age in thorax of flies by DR.  **C.** Venn diagram of numbers of significantly (p<0.01) changed metabolites at 10, 20, and 40 days of age in whole flies by DR.  **D.** Principle component analysis of thorax samples from all ages. Ellipses indicate 75% confidence interval. **E.** Principle component analysis of whole flies samples from all ages. Ellipses indicate 75% confidence interval

Figure S2: Effect of age and diet on the metabolome in C18 column separated metabolites. **A-C.** Metabolites that are significantly (FDR = 0.025) altered by age (blue circles), diet (green circles), or diet and age (orange circles) in the thorax at 10 days (A), 20 days (B), or 40 days (C) of age. **D-F.** Metabolites that are significantly (FDR = 0.025) altered by age (blue circles), diet (green circles), or diet and age (orange circles) in the whole fly at 10 days (A), 20 days (B), or 40 days (C) of age. The x-axis is the 40/10 day old ratio in metabolite concentration in AL fed flies and y-axis is the DR/AL ratio in metabolite concentration for the specific age measured, 10 days in (A) and 40 days in (B).

Figure S3: Pairs of thorax metabolites in whose mean values are unaffected by diet (p > 0.05) but whose correlations are significantly different between DR and AL. The upper plots show the correlation between two metabolites under AL (red) and DR (blue) conditions. The lower panels show the effect of diet on the absolute levels of each metabolite (none are statistically significant).

Figure S4 – **A.** Correlation between in overlapping metabolites from the targeted Metbolon approach and putatively matched metabolites by *mummichog* in the non-targeted high resolution approach (referred to as “Jones”) for the diet effect in 29 metabolites. The x-axis indicates the DR/AL in dataset run in the Jones lab, while the y-axis is the DR/AL ratio run on the Metabolon platform **B.** Examples of AL and DR levels of metabolites, kynerunine and acetylcarnitine, are shown in whisker plots to the right for both the metabolon and Jones data.
